# Linking ecosystem multifunctionality to microbial community features in rivers along a latitudinal gradient

**DOI:** 10.1128/msystems.00147-24

**Published:** 2024-03-06

**Authors:** Miaomiao Cai, Caifang Zhang, Caroline Njambi Ndungu, Guihua Liu, Wenzhi Liu, Quanfa Zhang

**Affiliations:** 1 CAS Key Laboratory of Aquatic Botany and Watershed Ecology, Wuhan Botanical Garden, Chinese Academy of Sciences, Wuhan, China; 2 Danjiangkou Wetland Ecosystem Field Scientific Observation and Research Station, Chinese Academy of Sciences & Hubei Province, Wuhan, China; 3 College of Life Sciences, University of Chinese Academy of Sciences, Beijing, China; University of Pretoria, Pretoria, South Africa

**Keywords:** ecosystem functioning, functional diversity, latitudinal pattern, network complexity, nitrogen cycling

## Abstract

Microorganisms regulate numerous ecosystem functions and show considerable differences along a latitudinal gradient. Although studies have revealed the latitudinal patterns of microbial community structure and single ecosystem function, the latitudinal patterns of ecosystem multifunctionality (EMF) and how microbial communities affect EMF along a latitudinal gradient remain unclear. Here, we collected channel sediments, riparian rhizosphere soils, and riparian bulk soils from 30 rivers across China and calculated EMF using 18 variables related to nitrogen cycling, nutrient pool, plant productivity, and water quality. We also determined microbial diversity (taxonomic and functional) and microbial network complexity using metagenomic sequencing. The results showed that EMF significantly decreased with increasing latitude in riparian rhizosphere and bulk soils but not in channel sediments. Microbial taxonomic and functional richness (observed species) in channel sediments were significantly higher in the low-latitude group than in the high-latitude group. However, microbial co-occurrence networks were more complex in the high-latitude group compared with the low-latitude group. Abiotic factors, primarily geographic and climatic factors, contributed more to EMF than microbial diversity and network complexity parameters in which only betweenness centralization had a significant relationship with EMF. Together, this study provides insight into the latitudinal pattern of EMF in rivers and highlights the importance of large-scale factors in explaining such latitudinal patterns.

**IMPORTANCE:**

Ecosystem multifunctionality (EMF) is the capacity of an ecosystem to provide multiple functions simultaneously. Microorganisms, as dominant drivers of belowground processes, have a profound effect on ecosystem functions. Although studies have revealed the latitudinal patterns of microbial community structure and single ecosystem function, the latitudinal patterns of EMF and how microbial communities affect EMF along a latitudinal gradient remain unclear. We collected channel sediments, riparian rhizosphere soils, and riparian bulk soils from 30 rivers along a latitudinal gradient across China and calculated EMF using 18 variables related to nitrogen cycling, nutrient pool, plant productivity, and water quality. This study fills a critical knowledge gap regarding the latitudinal patterns and drivers of EMF in river ecosystems and gives new insights into how microbial diversity and network complexity affect EMF from a metagenomic perspective.

## INTRODUCTION

The capacity of an ecosystem to provide multiple functions simultaneously, that is ecosystem multifunctionality (EMF), has received increasing attention in recent years (1–4). Exploring the spatial pattern of EMF based on multiple functions (e.g., plant productivity, biogeochemical cycling, and nutrient pools) can provide a comprehensive understanding of ecosystem services under global environmental change (5). The EMF is influenced by a wide range of environmental and biological factors, such as geographic location, climate, edaphic properties, and microbial communities (6, 7). Examining the relationships between such diverse factors and EMF is essential for predicting the response of EMF to rapidly increasing anthropogenic activities.

Belowground microorganisms, including archaea, bacteria, fungi, and viruses, are highly diverse and complex (8, 9). They are proxies of soil microbial communities and harbor various functional features, thus supporting multiple ecosystem functions simultaneously, such as plant primary production and nutrient cycling (10, 11). The fact that bacterial or fungal diversity has a profound effect on ecosystem functions has been evidenced recently (6, 12–14). However, the roles of multidimensional diversity (e.g., taxonomic and functional) of microbial communities in driving EMF have been less investigated, hampering our capacity to fully understand the associations between biodiversity and ecosystem functioning.

Soil microorganisms are interconnected through information, energy, and materials exchanges, forming a complex ecological network, which is critical for regulating ecosystem processes and functioning (9, 15). Recently, co-occurrence network analysis has been widely used to reveal microbial interactions within a community based on environmental heterogeneity and resource availability (10, 14, 16). Topological parameters of co-occurrence networks, such as node number, edge number, betweenness centralization, and clustering coefficients, are usually used to characterize the microbial network complexity (17, 18). However, the relationships between microbial network complexity and EMF remain largely unknown, especially at a large spatial scale.

As a typical model system in ecology, the latitudinal gradient exhibits great differences in climate, soil physicochemical properties, and biological communities over long geographic distances (19, 20). For instance, the temperature and precipitation are significantly higher in the south and lower in the north of the Qinling–Huaihe Line across China (21). Plants and animals generally show monotonically decreasing or hump-shaped diversity patterns with increasing latitude (22). However, several studies have demonstrated that microorganisms do not follow the latitudinal diversity patterns of macroorganisms (23–25), which drive ecosystem functioning. Therefore, advancing our understanding of the changes in microbial diversity along a latitudinal gradient is helpful to reveal the influence of microbial communities on EMF (12, 26, 27).

River ecosystems harbor high microbial diversity and provide a wide range of ecosystem functions and services. However, most related studies only focus on a single function, such as nitrogen removal (28), and EMF has been less explored in rivers (29, 30). In the present study, we collected channel sediments, riparian rhizosphere soils, and riparian bulk soils from 30 rivers along a latitudinal gradient in China and calculated EMF using 18 variables which together constitute a good proxy for nitrogen cycling, nutrient pools, plant productivity, and water quality. We also determined microbial diversity (taxonomic and functional) and microbial network complexity by metagenomic sequencing. We aimed to (1) investigate the latitudinal patterns of EMF, microbial diversity, and microbial network complexity in rivers (2), elucidate the relationships of microbial diversity and network complexity with EMF, and (3) identify the driving factors of EMF along a latitudinal gradient.

## MATERIALS AND METHODS

### Study sites and sampling

In September 2018, 30 higher-order rivers were selected for soil, sediment, and water sampling along a latitudinal gradient across eastern China (Fig. S1), including the Songhua River, Yellow River, and Yangtze River. We established a representative sampling site for each river in the middle and lower reaches, where riparian areas form relatively wide bands and are flat in topography. The latitude of sampling sites varies between 21.76° N and 45.09° N, while the elevation ranges from −1.4 m to 1000.2 m (Table S1). These sampling sites were separated into low-latitude (N = 11) and high-latitude (N = 19) groups according to the Qinling Mountains–Huaihe River Line (i.e., Qinling–Huaihe Line), which is the geographical and climatic boundaries between the south and north China (21).

At each sampling site, channel sediments (0–20 cm), riparian rhizosphere soils (0–20 cm), and riparian bulk soils (0–20 cm) were collected. Specifically, for sediment collection, approximately 200 g of surface sediments were randomly taken from three channel sites at a distance of 5–10 m from the shore using a homemade grab sampler and mixed thoroughly to form a composite sample. For collecting riparian rhizosphere soils, the plants within a riparian plot (1 × 1 m) established in the representative plant community were excavated using a spade at a depth of 20 cm. After shaking off the soils loosely attached to the roots, the remaining soils tightly bound to the roots (i.e., rhizosphere soils) were collected with a sanitized soft brush (31, 32). Bulk soils were collected from three bare sites adjacent to the vegetation plot using a soil drilling machine (Zhonghe Technology, Beijing, China) (33, 34). The areas for collecting rhizosphere and bulk soils were generally 0.5‒2 m above the river water level and at least 5 m from the water. The sampled sediments and soils were each separated into two parts. One part was kept in a portable refrigerator for measuring physicochemical properties, and the other part was frozen in liquid N_2_ for microorganism analysis. In addition, a 2 L surface water sample was collected at 0.5 m below the water surface in each site to determine water physicochemical properties. All plants in each riparian plot were dug out carefully, and after cleaning, the total fresh biomass of all plants was weighed using a portable electric balance on the spot.

### Measurement of soil, sediment, and water physicochemical properties

Potential nitrification (PN) rates were measured using the shaken soil/sediment slurry method, as described previously (28). The denitrification (DN) and anammox (AN) rates of soil/sediment were determined using the ^15^N isotope pairing technique (4). Briefly, 5 g homogenized fresh soils were incubated in 12 mL glass vials (Exetainer, Labco, High Wycombe) with anaerobic distilled water. After pre-incubation on a horizontal shaker at 25°C for 72 h, the residual O_2_ and background NO_3_^−^ and NO_2_^−^ were depleted. Subsequently, these vials were divided into three groups and injected with 100 µL ^15^N containing solutions (^15^NH_4_Cl, ^15^NH_4_Cl + KNO_3_, and K^15^NO_3_), resulting in a final ^15^N concentration of 100 µmol/L in each vial, and then incubated at 25°C. Finally, 200 µL of 7 mol/L ZnCl_2_ solution was injected into each vial to terminate the reaction at 0 and 8 h. The concentrations of the produced ^29^N_2_ and ^30^N_2_ were determined using isotope ratio mass spectrometry (Gasbench II and Delta V Advantage, Thermo Finnigan, Germany) and used for the denitrification and anammox rates calculation.

Soil pH and electrical conductivity were determined by a pH/conductivity meter with a soil-to-water ratio of 1:5 (vol/vol), while soil moisture content was measured gravimetrically after 10 g of fresh soil drying to constant weight at 105°C. Approximately 50 g of air-dried soil was sieved through a 10-mesh sieve to determine the soil texture (i.e., the percentage of sand, silt, and clay). The soil density was analyzed by 15 g soil after drying overnight at 105°C using the specific gravity method. The NH_4_^+^ and NO_3_^–^ concentrations of soil/sediment were measured by extracting 10 g of fresh soil/sediment with 100 mL of 2 mol/L KCl, filtering, and running a colorimetric analysis of the filtrate using an automated nutrient analyzer (EasyChem plus, Systea, Italy). The total carbon (TC), organic carbon (TOC), and total nitrogen (TN) concentrations of air-dried soil/sediment samples were measured using an elemental analyzer (Vario TOC cube, Hanau, Germany). Total phosphorus (TP) content in soil/sediment was measured by the molybdenum blue method with a spectrophotometer (Shimadzu UV-1800, Tokyo, Japan) after digestion (35).

Water temperature, pH, dissolved oxygen (DO), and total dissolved solids (TDS) were determined *in situ* with a YSI Professional Plus multi-parameter water quality meter (YSI Inc., Ohio, USA). The TOC and TN contents of water were determined using the TOC/TN analyzer (Vario TOC cube, Hanau, Germany). Water NH_4_^+^ and NO_3_^−^ concentrations were determined by an automated nutrient analyzer (EasyChem plus, Systea, Italy). Water TP was analyzed by an acid digest followed by the colorimetric assay with the molybdenum blue method (36). The chlorophyll-a (Chla) concentration was measured by filtering 500 mL of water through 0.45 µm glass microfiber filters, followed by extraction with 90% acetone and measurement with a spectrophotometer at λ = 665 nm and 649 nm (Beckman, Fullerton, USA). All abbreviations of the soil, sediment, and water physicochemical properties are listed in Table S2.

### Assessment of EMF

We used 18 variables related to nitrogen cycling, nutrient pools, plant productivity, and water quality to calculate EMF (Fig. S2). For nitrogen cycling, we focused on the rates of nitrification, denitrification, and anammox, which are the dominant processes supporting the nitrogen removal function of river ecosystems. TC, TOC, TN, NO_3_^–^, NH_4_^+^, and TP contents in soil/sediment were regarded as nutrient pools. Plant productivity was represented by a single function, that is, plant biomass. Water DO, TDS, TOC, TN, NH_4_^+^, NO_3_^−^, TP, and Chla contents were considered as the proxies for water quality.

The original data were multiplied by −1 to maintain directional change when proxies represent an undesirable environmental perspective, including water TDS, TOC, TN, NH_4_^+^, NO_3_^−^, TP, and Chla contents (3). Furthermore, to maintain comparability, the 18 variables were standardized to a common scale (0–1) according to the following formula: STD = (X − X_min_)/(X_max_ − X_min_), where STD is the standardized variable, X is the target variable, X_min_ is the minimum value, and X_max_ is the maximum value across all samples. For calculating the multifunctionality index, four independent multifunctionality approaches were used: (i) multiple single functions, (ii) average multifunctionality index (EMF_average_), (iii) weighted multifunctionality index (EMF_weighted_), and (iv) multiple-threshold multifunctionality index (37, 38). The EMF_average_ was calculated by simply averaging the STD of all individual functional variables equally. For calculating EMF_weighted_, the functions within the same functional group (i.e., nitrogen cycling, nutrient pools, plant productivity, and water quality) were weighted equally to avoid overweighting certain aspects of ecosystem functioning and assessment bias because of the overrepresentation of related functions (39, 40). The EMF_weighted_ was calculated using the weighted average method, with each category of ecological function given equal weight. The multi-threshold multifunctionality index was calculated as the number of functions that simultaneously exceeded threshold values between 5% and 95% at 1% intervals of the maximum for given functions (37). Each multifunctionality approach has pros and cons. For example, the single-function method helps identify which function drives multifunctionality, but it struggles to assess the overall function of ecosystems and fails to consider the relationships between functions (12). Currently, the average method is widely used due to its simplicity and ease of interpretation (16, 18, 41). It can provide a clear evaluation of the average effect of variables on multiple ecosystem functions. However, this method ignores the trade-offs and synergies of functions, which can lead to biased results (3, 39). Although the weights between functions are taken into account when using the weighted average method, the weights of functions are not easy to measure and are difficult to widely promote and use (39, 40). The multiple threshold method is useful in examining the relationship between the variables and the number of functions that reach a specific threshold, and thus identifying the turning point where the relationship changes. However, this method has a drawback as it does not consider the correlation and importance among functions (37). Hence, the use of multiple methods allows us to maintain the comparability of the results with other works (14, 38).

### Microbial sequencing and metagenome assembly

In this study, 0.5 g soil/sediment was used to extract genomic DNA using the PowerSoil DNA Isolation kit (MoBio, California, USA) following the standard protocol. The qualified DNA samples were fragmented by sonication to a size of 350 bp, and DNA fragments were then end-polished, A-tailed, ligated with full-length adapters, and purified. After library construction, the confirmed high-quality genomic DNA was used for metagenome sequencing on the Illumina NovaSeq 6000 platform (Illumina, San Diego, USA). Finally, the adapters and low-quality sequences, including reads with a high N base content (up to 10% of the read length) and reads with low-quality bases (quality values ≤10, exceeding 50% of the read length), were processed using Trimmomatic v0.39, resulting in approximately 6 Gb of raw data for each sample.

The metagenome assembly for clean reads was performed with MEGAHIT v1.1.2 to obtain contigs longer than 500 bp. MetaGeneMark v3.25 was used for predicting the open reading frame (ORF) of all contigs, and CD-HIT v4.6.8 was employed to cluster ORFs with 95% identity and coverage >90%, resulting in a non-redundant ORF set (42). Regarding taxonomic annotation, the ORFs were searched against the proGenomes database using Kaiju v1.6.3 with default parameters. For function annotation, the non-redundant genes were screened in the Kyoto Encyclopedia of Genes and Genomes (KEGG) database using BLAST v2.2.21 to obtain the KEGG Orthology number and pathway annotation information (42).

### Analysis of microbial diversity and co-occurrence network

The α-diversity of the microbial community (species level) and functional genes (KEGG Orthology), including observed species and Shannon indexes, were calculated using the package “vegan” v2.5-7 in R v4.2.2. For evaluating the β-diversity of the microbial community (species level) and functional genes (KEGG Orthology), non-metric multidimensional scaling (NMDS) was constructed according to the Bray–Curtis dissimilarities and NMDS1 and NMDS2 were used to represent the β-diversity of microbial community and functional genes.

For evaluating the microbial network complexity, we constructed microbial co-occurrence networks based on the Spearman correlation matrix with the package “Hmisc” in R v4.2.2. A total of 3,406 genera were obtained to construct the networks of the low-latitude and high-latitude groups in rhizosphere soil, bulk soil, and sediment, respectively. According to the previous works (6, 14), only robust correlations with correlation coefficients >0.65 and false discovery rate‐adjusted *P* values <0.01 were kept in the final co-occurrence networks. In addition, for subnetwork analysis of each sample, microbial genera that had a sum relative abundance <0.01% in the soil samples were removed. Ten network topological characteristics of each sample were extracted using the subgraph function via package “igraph” in R v4.2.2 to assess microbial network complexity (8, 16). Specifically, node number represents the number of genera, and edge number is the number of connections among all of the nodes. Betweenness centralization is the number of times a node acts as a bridge along the shortest path between two other nodes, and degree represents the extent of nodes in a network associated with other nodes in the network. Average path length indicates the average network distance between all pairs of nodes, and connectance represents the proportion of realized links from all possible connections in the network. Diameter is the greatest distance between the nodes that exist in the network, and edge connectivity represents the smallest number of edges whose removal disconnects. Degree centralization represents the number of connections across the node, and the clustering coefficient is the degree to which the nodes tend to cluster together.

### Statistical analyses

Upon conducting normality testing of the raw data, we discovered that some of the data did not adhere to normal distribution and proved to be challenging to convert. Hence, in the data analysis for this article, we opted to utilize non-parametric analysis methods to maintain consistency in data statistics. The Mann-Whitney U test was used to evaluate the statistical differences in single ecosystem function, EMF, and microbial α-diversity indices in channel sediments, riparian rhizosphere soils, and riparian bulk soils between low-latitude and high-latitude groups. Spearman correlation analyses were conducted to construct a correlation matrix of 18 single functional variables and link them to latitude using the package “ggcor.” Furthermore, the linear regression models were constructed to depict the linear relationship between EMF and latitude. The drivers of community composition were assessed using distance-based redundancy analysis (dbRDA) with Bray–Curtis dissimilarities via package “vegan” v2.5-7. Furthermore, the generalized additive model (GAM) was supplemented to reveal the relationship between Bray–Curtis dissimilarities and latitude via package “vegan” v2.5-7 and “metR.” Meanwhile, the analysis of similarities (ANOSIM) was performed to test inter-group differences based on the Bray–Curtis distance using the package “vegan” v2.5-7 in R 4.2.2. In addition, Spearman correlation analyses were used to link the single functional variables and EMF to environmental factors (geographic, climatic, and edaphic factors), microbial diversity, and network complexity. The visualization of the heatmap was carried out using the package “pheatmap.” Based on Spearman correlation analyses, EMF-related factors were selected for random forest analysis to identify the main factors that best explained the spatial variation in the EMF using the package “randomForest.” The importance and significance of predicted factors were assessed by the percentage increase in mean squared error (%IncMSE) using the package “rfPermute.” The significance of the model was assessed with 1,000 permutations of the response variable using the package “A3.” Partial least squares path modeling (PLS-PM) was applied to elucidate the direct and indirect effects of factor groups (i.e., geographic location, climate, edaphic factors, habitats, microbial diversity, and network complexity) on EMF. Here, after performing principal component analysis, the most representative axis 1 was selected to represent network complexity (14). Before calculating the index, the average path length and diameter were multiplied by −1 to maintain directional change as they represent the network sparsity. All statistical analyses were performed in R 4.2.2.

## RESULTS

### Latitudinal patterns of individual ecosystem functions and multifunctionality

**Fig 1:**
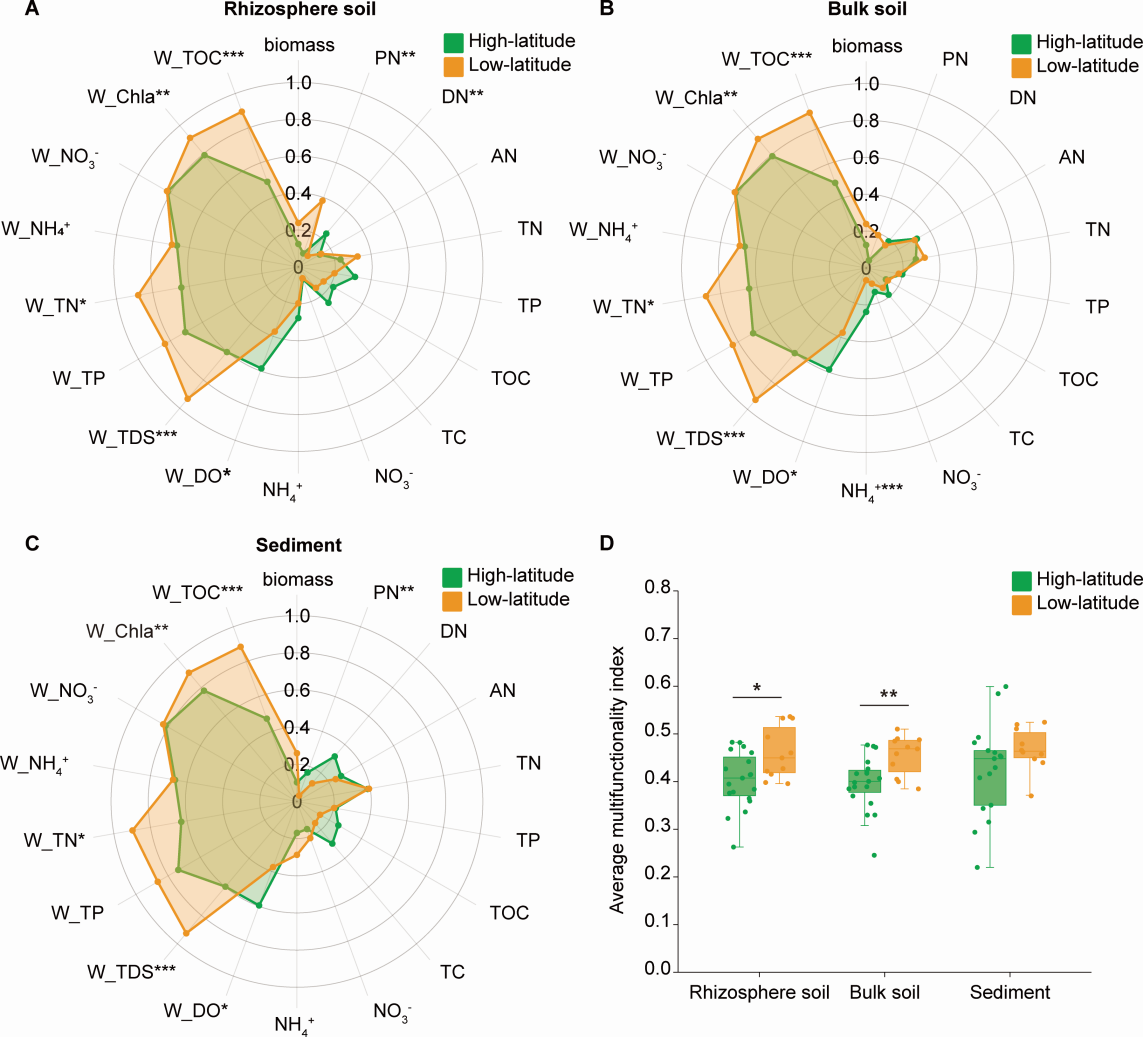
The difference in ecosystem functions and average multifunctionality index (EMF_average_) in rhizosphere soil (**A, D**), bulk soil (**B, D**), and sediment (**C, D**) between the high-latitude and low-latitude groups. *, **, and *** indicate *P* < 0.05, 0.01, and 0.001, respectively. PN, potential nitrification rate of soil/sediment; DN, denitrification rate of soil/sediment; AN, anammox rate of soil/sediment; TC, total carbon content of soil/sediment; TOC, total organic carbon content of soil/sediment; NO_3_^−^, nitrate content of soil/sediment; NH_4_^+^, ammonium content of soil/sediment; TN, total nitrogen content of soil/sediment; TP, total phosphorus content of soil/sediment; W_DO, dissolved oxygen of water; W_TDS, total dissolved solids of water; W_TOC, total organic carbon of water; W_TN, total nitrogen content of water; W_NH_4_^+^, ammonium content of water; W_NO_3_^−^, nitrate content of water; W_TP, total phosphorus content of water; W_Chla: chlorophyll a of water.

The differences in single functions between the low-latitude and high-latitude groups are displayed in Fig. 1; Table S3. In rhizosphere soil, the PN rate was significantly higher in the low-latitude group, but the denitrification rate was higher in the high-latitude group (Fig. 1B). In bulk soil, soil NH_4_^+^ was enriched in the high-latitude group (Fig. 1B). In sediment, the PN rate was significantly higher in the high-latitude group (Fig. 1C). Moreover, the water samples in the low-latitude group had lower DO and higher TDS, TOC, TN, and Chla (Fig. 1A through C).

**Fig 2:**
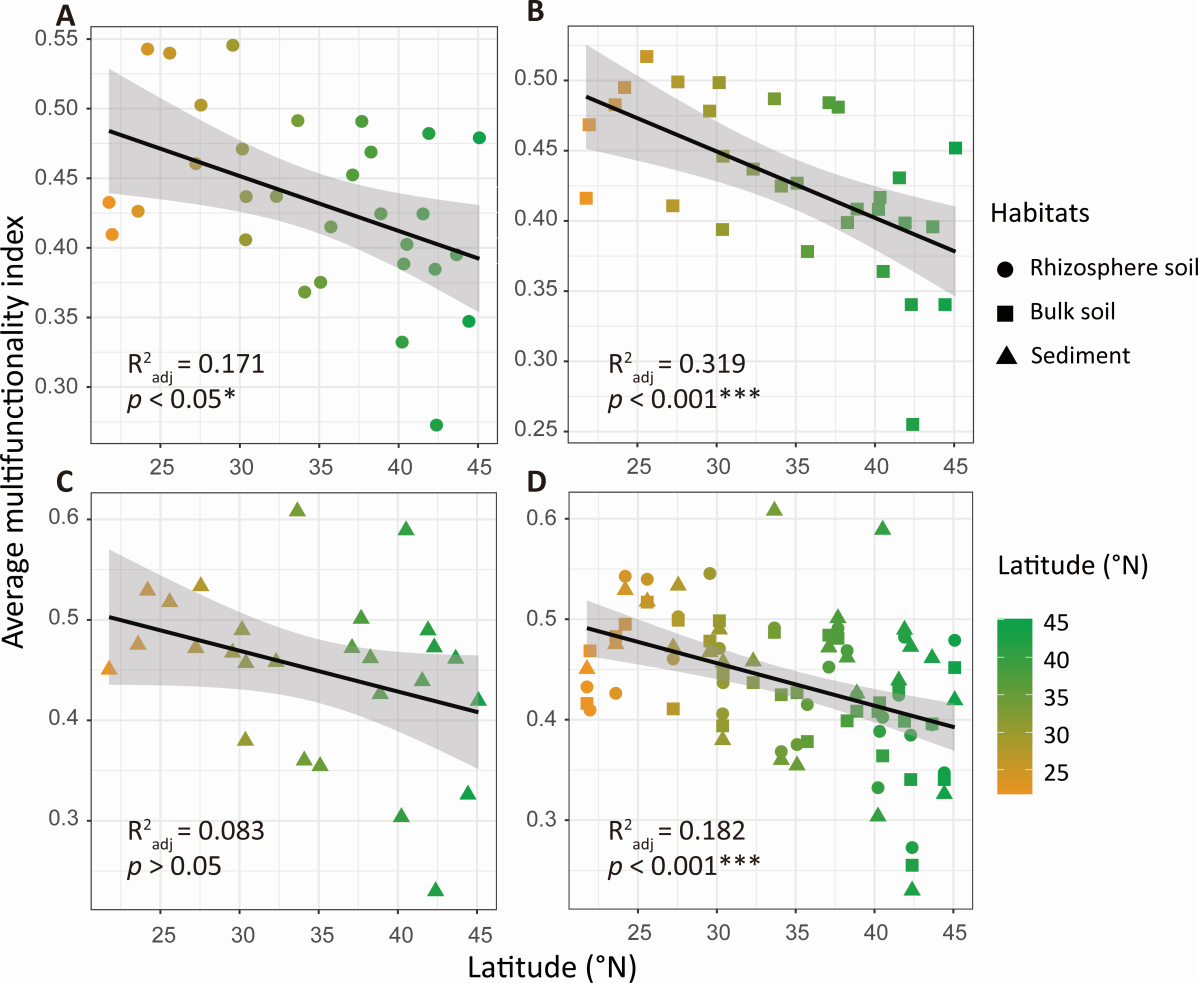
Liner regression model describing the relationship between latitude and average multifunctionality index (EMF_average_) among habitats. (A) Rhizosphere soil; (B) bulk soil; (C) sediment; and (D) all samples.

**Fig 3:**
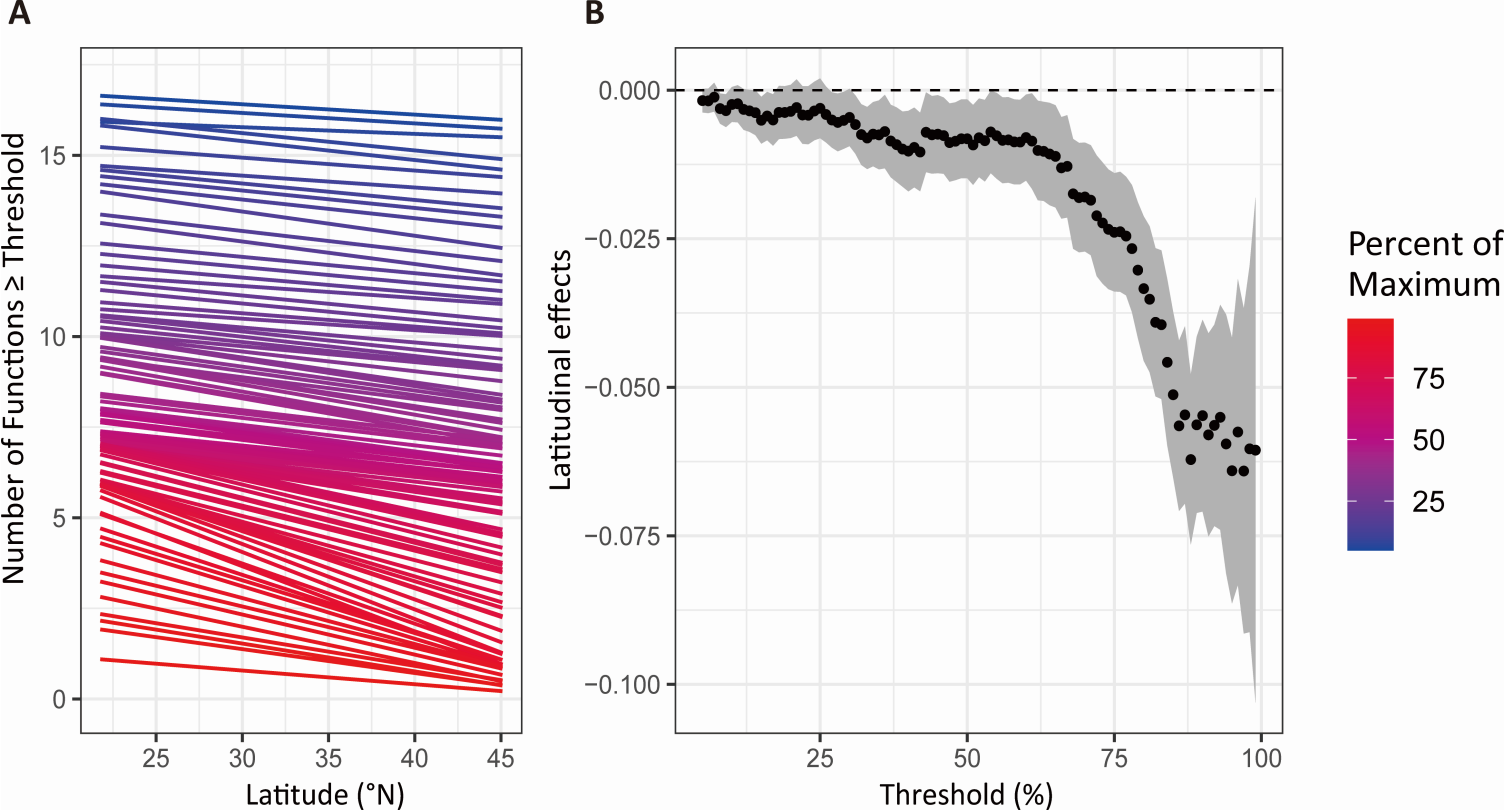
Latitudinal effects on EMF through multiple thresholds approach. (**A**) The relationship between the number of functions exceeding a threshold level from 5% and 95% of maximum functioning (i.e., multi-threshold multifunctionality index) and latitude. Different colors represent different thresholds of functioning. The curve shows the slope changes of each colored line with threshold levels. (**B**) The black points and shadowed area indicate the slope and the 95% confidence interval.

In riparian rhizosphere and bulk soils, EMF_average_ and EMF_weighted_ were significantly higher in the low-latitude group compared with the high-latitude group (Fig. 1D; Fig. S3). However, we found no significant difference in sediment EMF_average_ and EMF_weighted_ between the low-latitude and high-latitude groups (Fig. 1D; Fig. S3). Additionally, latitude had significant negative associations with EMF (Fig. S4). EMF_average_ and EMF_weighted_ significantly decreased with increasing latitude in riparian rhizosphere and bulk soils but not in channel sediments (Fig. 2; Fig. S5). The generalized linear mixed model also showed that EMF calculated by the multiple-threshold method was negatively associated with latitude (Fig. 3; Fig. S6).

### Latitudinal patterns of microbial diversity and network complexity

**Fig 4:**
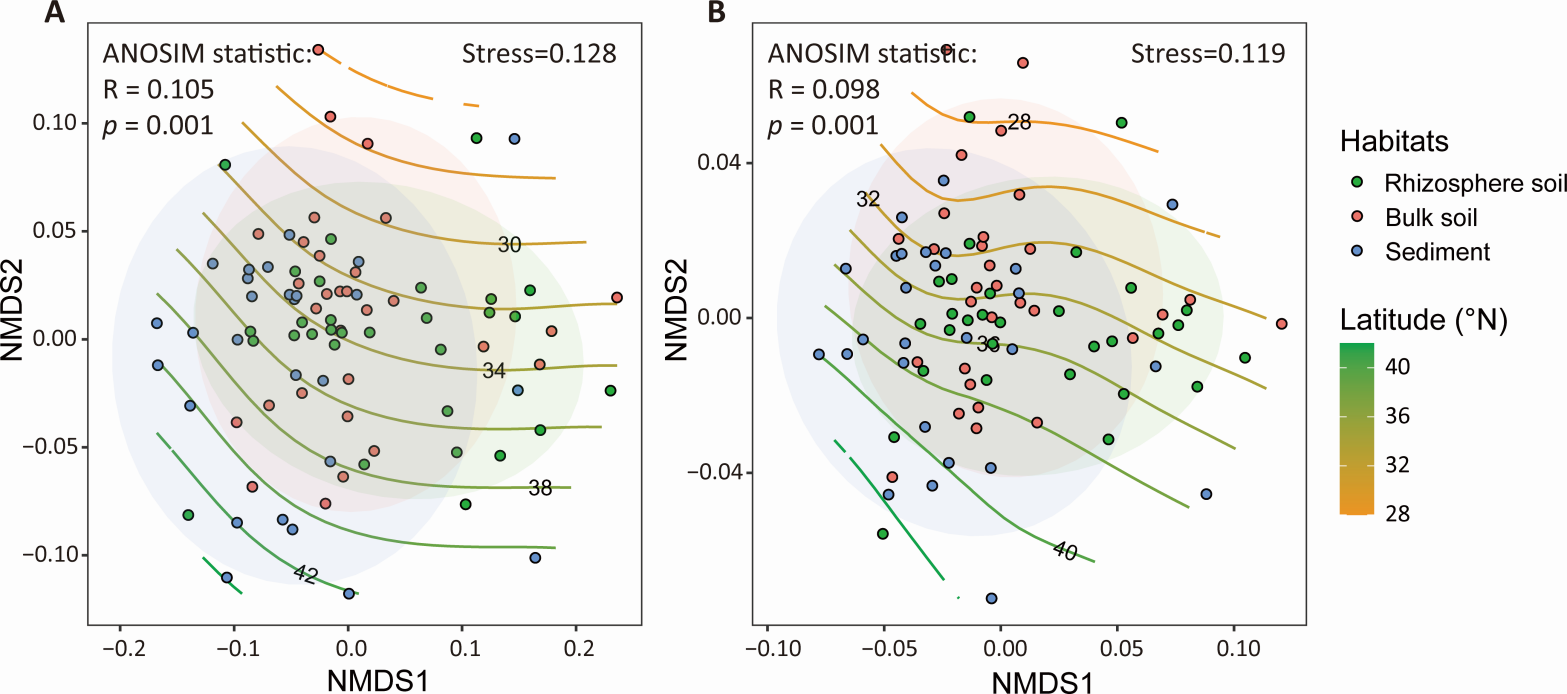
NMDS ordination based on Bray–Curtis dissimilarities along a latitudinal gradient with the GAM. (**A**) Microbial community (species level) separated by habitats and (**B**) functional genes (KEGG Orthology) separated by habitats.

For microbial α-diversity, microbial taxonomic and functional richness (i.e., observed species) in channel sediments was significantly higher in the low-latitude group than in the high-latitude group (Table S4). Moreover, the Shannon index of functional genes was higher in the high-latitude group in both riparian bulk soil and channel sediments (Table S4). For microbial β-diversity, latitude, clay, and pH were identified as the dominant drivers of microbial community and functional gene composition based on dbRDA results (Fig. S7). NMDS ordination also showed significant variations in the microbiota and functional profiles among habitats with *P* < 0.001 (ANOSIM statistic) along a latitudinal gradient (Fig. 4). Spearman correlation analyses indicated that latitude was positively correlated with the Shannon index of functional genes but negatively related to NMDS2 of microbial community and functional genes among all samples (Fig. S8).

**Fig 5:**
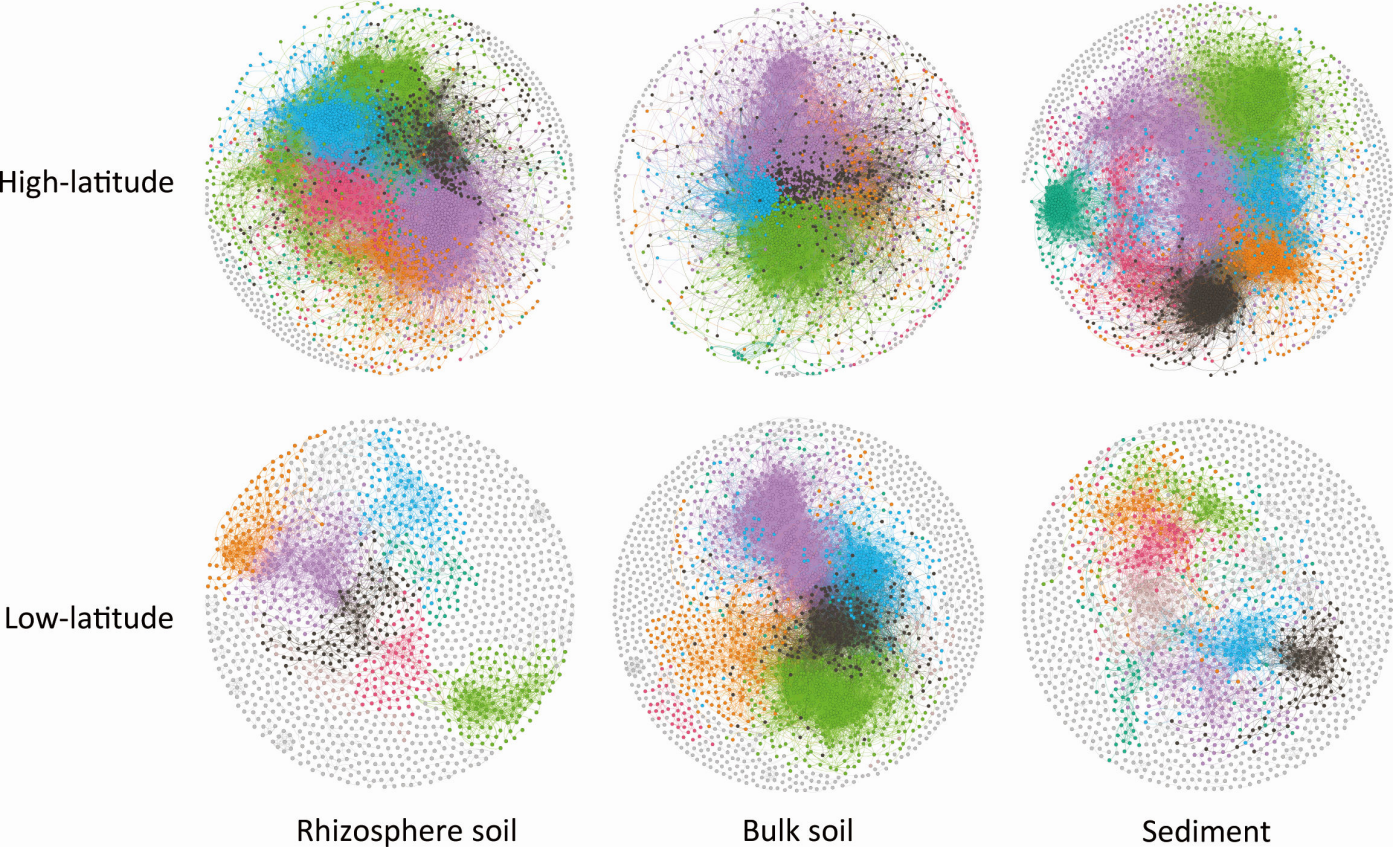
The microbial co-occurrence subnetworks of different habitats in high-latitude and low-latitude groups. The nodes represent the main genera of microorganisms, and the connections indicate strong and significant correlations. Networks are randomly colored by modules.

Microbial co-occurrence networks of different habitats were constructed to evaluate the difference in network complexity between low-latitude and high-latitude groups (Fig. 5). There were 2,819, 2,825, and 3,054 nodes and 892,779, 385,389, and 342,938 links in co-occurrence networks of rhizosphere soil, bulk soil, and sediment, respectively. In all three habitats, the high-latitude networks were found to have higher values of nodes, edges, and clustering coefficient, and lower values of average path length and modularity (Table S5), implying that microbial co-occurrence networks were more complex in the high-latitude group compared with the low-latitude group. Modularity values were greater than 0.4 (except for the rhizosphere soil network in the high-latitude group), indicating that the constructed networks had modular structures (Table S5). Among all topological features of subnetworks, only clustering_coefficient was different between the high-latitude group and low-latitude group in bulk soil (Table S6). No significant relationship was found between topological features of co-occurrence networks and latitude (Fig. S8).

### Driving factors of EMF along a latitudinal gradient

Significant correlations were observed between EMF and geographic and climate factors, including longitude, latitude, mean annual temperature (MAT), and mean annual precipitation (MAP) (Fig. S9). For edaphic factors, soil pH was negatively correlated with EMF_average_ and EMF_weighted_, and the percentage of fine substrates (i.e., clay and silt) were positively correlated with EMF_weighted_ (Fig. S9). For microbial diversity and network complexity, only betweenness centralization was negatively correlated with EMF_average_, and microbial diversity has no significant relationship with EMF. Significantly, the relationships between EMF and environmental and microbial variables varied among rhizosphere soil, bulk soil, and sediment (Fig. S10 to S12).

**Fig 6:**
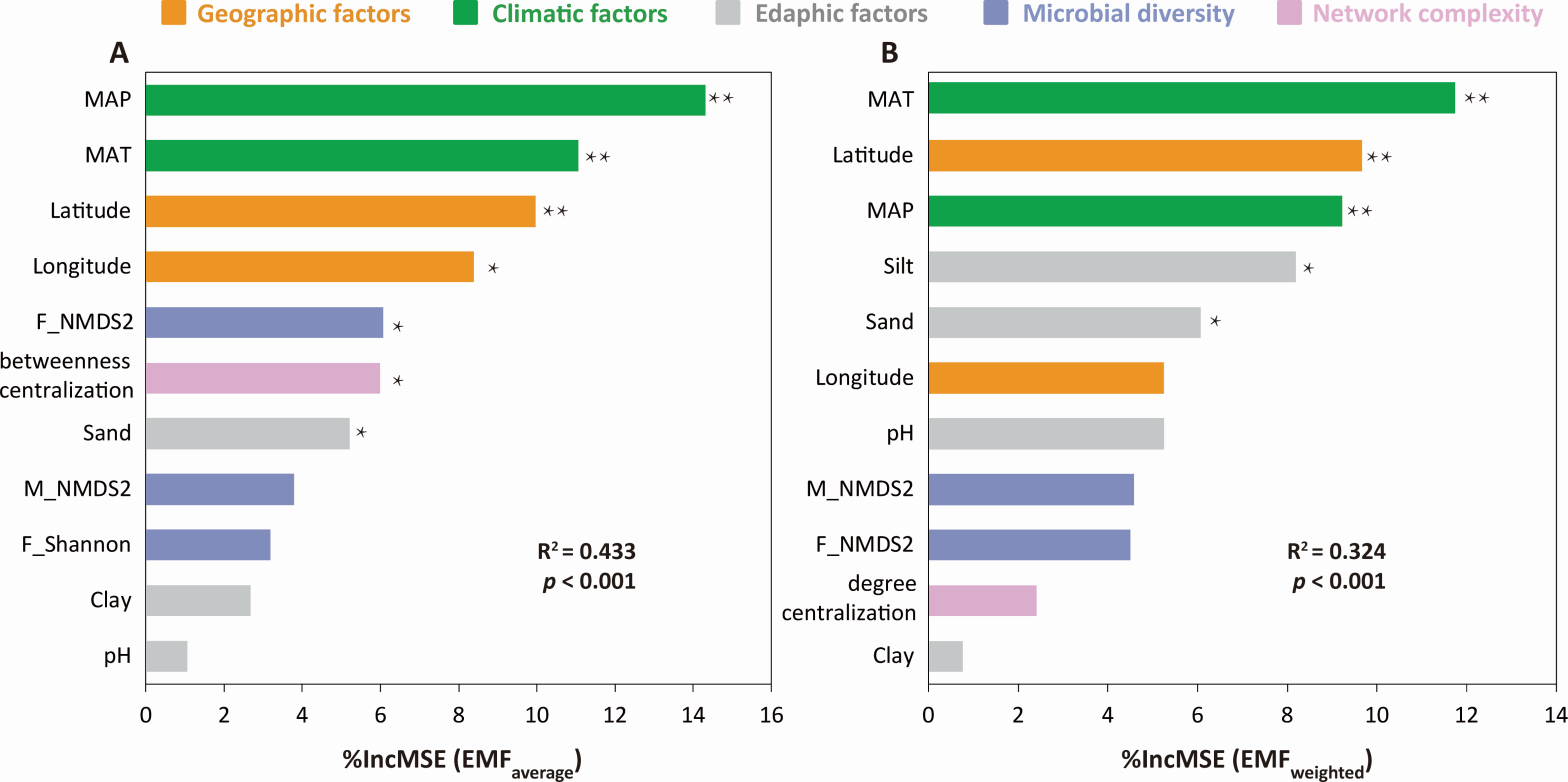
The random forest analyses depicting the importance of predictive factors in explaining EMF. (A) EMF_average_ and (B) EMF_weighted_. * and ** indicate *P* < 0.05 and 0.01, respectively. R^2^ indicates the proportion of variance explained.

**Fig 7:**
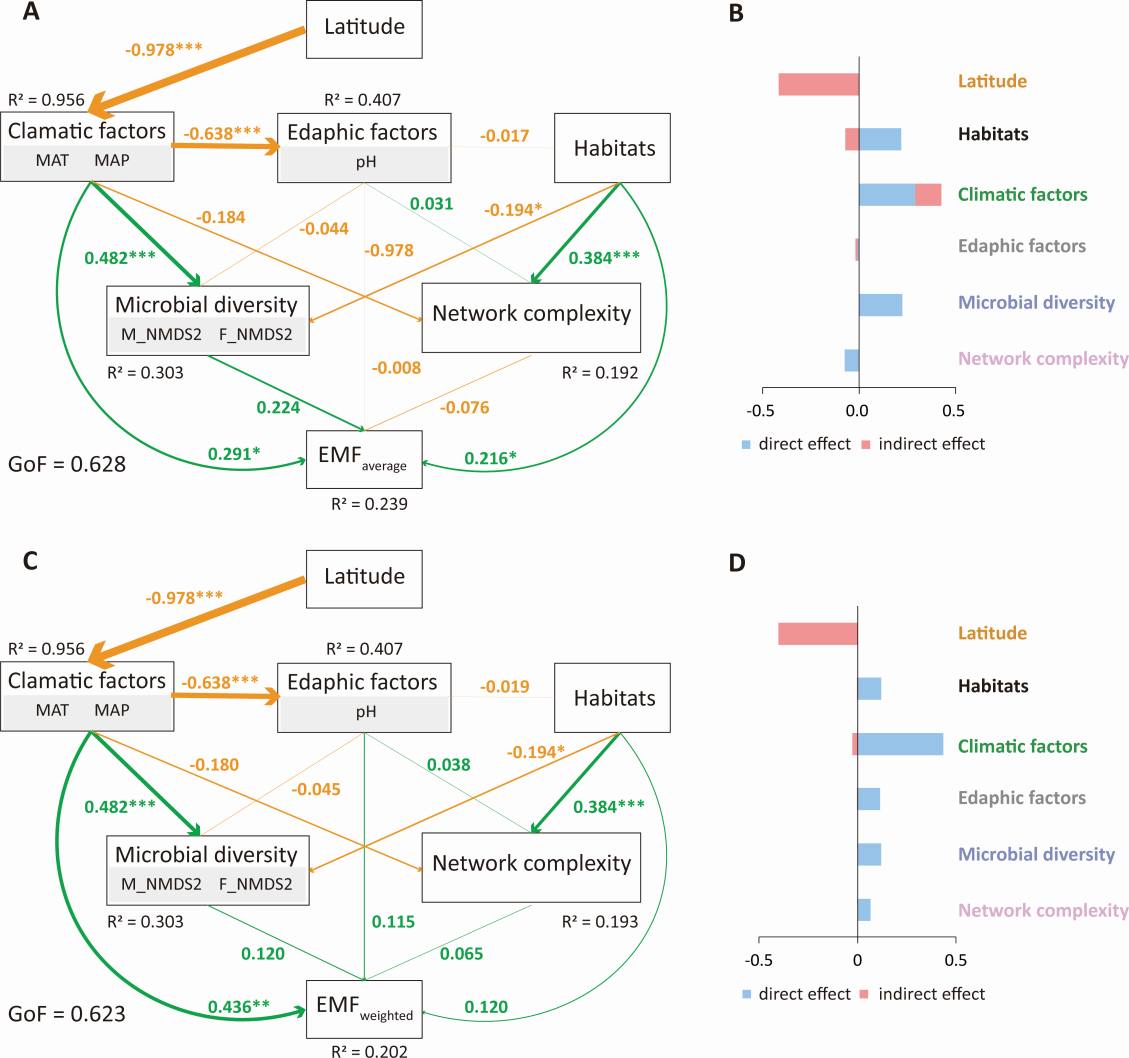
Partial least squares path model showing the cascading effects of environmental and microbial factors on EMF_average_ (**A, B**) and EMF_weighted_ (**C, D**). Numbers adjacent to the arrows are standardized path coefficients (** *P* < 0.01, *** *P* < 0.001). Solid green lines indicate significantly positive effects and solid orange lines indicate significantly negative effects. The dotted lines indicate non-significant effects. The R^2^ value is the total variation explained by the model. Direct effects are equal to the path coefficients, while indirect effects refer to the mathematical product of all possible paths.

Random forest analysis showed that MAP contributed most (14.30%) to the variance in EMF_average_, followed by MAT, latitude, and longitude (Fig. 6A). Moreover, F_NMDS2 and betweenness centralization also played an important role in explaining the EMF_average_ (Fig. 6A). For the EMF_weighted_, climate (MAT and MAP) and geographic factors (latitude and longitude) contributed 35.22% and 14.88% of the variance in EMF_weighted_, respectively (Fig. 6B). In addition, the percentage of silt and sand accounted for 14.20% of the EMF_weighted_ variance. The PLS-PM demonstrated that latitude was the most important factor influencing EMF_average_ and EMF_weighted_, and this factor regulated the EMF indirectly through its effects on climate (Fig. 7). Moreover, habitats (i.e., rhizosphere soil, bulk soil, and sediment) and microbial diversity had a considerable total effect on the EMF_average_ (Fig. 7B). The final model, which included a subset of the direct and indirect effects, together accounted for 23.9% and 20.2% of the variation in EMF_average_ (GoF (Goodness-of-Fit) = 0.628) and EMF_weighted_ (GoF = 0.623), respectively (Fig. 7).

## DISCUSSION

### Ecosystem functions and multifunctionality along a latitudinal gradient

Soil and sediment support a number of important ecosystem functions, including plant growth, nutrient cycling, water purification, and climate regulation (28). However, a critical knowledge gap regarding the geographical pattern of ecosystem functions remains to be addressed in the coming years as research questions and technologies continue to evolve. In the present study, we found that individual ecosystem functions in channel sediments and riparian rhizosphere soils, such as PN rate, showed diverse distribution patterns along a latitudinal gradient (Fig. 1). According to a global synthesis conducted by Li et al. (43), the soil nitrification rate tended to increase with decreasing latitude across terrestrial ecosystems, especially in the Northern Hemisphere, consistent with our results found in rhizosphere soil (Fig. 1A). In contrast, we found that most water quality parameters declined with decreasing latitude due to the higher water TDS, TN, TP, and Chla and lower DO in the low-latitude group (Fig. 1). These results imply that the latitudinal pattern of single ecosystem function in river ecosystems is diverse and complex and shaped by different mechanisms.

Using single ecosystem functions as a proxy for the overall functioning of an ecosystem generally neglects other vital functions and the trade-offs and synergies among functions (39). In this study, the average method was used to explore the relationship between EMF and latitude, and we found that the EMF_average_ significantly decreased with increasing latitude in the riparian rhizosphere and bulk soils but not in channel sediments across eastern China, as well as EMF_weighted_, which calculated using weighted average method (Fig. 1 and 2; Fig. S3). Moreover, the generalized linear mixed model showed that EMF was negatively associated with latitude when using a multiple-threshold approach (Fig. 3). Although the latitudinal patterns of EMF have not been extensively studied, previous research has shown that climatic factors are closely related to EMF. For example, Yan et al. (41) found that precipitation during the growing season was positively associated with EMF in 840 vegetation plots across a large climatic gradient in China, and Jiao et al. (14) reported that mean annual temperature had a positive relationship with EMF in 228 agricultural fields in China. Therefore, the effects of latitude on EMF revealed by the present study may be mainly mediated through changes in temperature and precipitation, which are vital factors driving ecosystem processes, thus regulating ecosystem functions.

### Response of microbial diversity and network complexity to latitude changes

Microorganisms in soil and sediment are extremely abundant and diverse and play a crucial role in supporting the multifunctionality of an ecosystem (9). However, we know relatively little about how microbial diversity responds to latitude changes due to limited technology in determining diversity accurately in the past (44, 45). In this study, using metagenomic sequencing, we found that microbial taxonomic and functional richness was significantly higher in the low-latitude group than in the high-latitude group in channel sediments but not in riparian soils (Table S4), consistent with the typical latitudinal diversity gradient (LDG). Moreover, latitude dominantly drove microbial community and functional gene dissimilarities (Fig. 4; Fig. S7). A variety of ecological and evolutionary theories, including the geographic area hypothesis, productivity hypothesis, evolutionary speed hypothesis, and ambient energy hypothesis, have been proposed to account for the LDG (22). However, numerous studies have shown that soil microorganisms deviate from the LDG and exhibit diversity patterns, including hump-shaped, reverse, or even indistinct diversity patterns along a latitudinal gradient (19). For example, based on metagenomics data of global topsoil samples, Bahram et al. (23) reported that both taxonomic and functional gene diversity of soil bacteria peaked at mid-latitudes and declined toward the equator and the poles. These results imply that the response of microbial diversity to latitude changes is highly complex and may vary with microbial taxa, ecosystem types, seasons, and spatial scales.

Recently, the microbial network complexity has attracted increasing attention in terms of biogeography and microbial ecology (9, 10). However, the latitudinal patterns of microbial network complexity remain virtually unknown, hampering our capacity to fully understand the influences of microbial communities on ecosystem functions and multifunctionality. In the present study, we found that microbial network complexity parameters had no significant relationship with latitude (Fig. S8). However, when we separated the sampling sites into the low-latitude and high-latitude groups and constructed six subnetworks (Fig. 5), the results showed that the low-latitude subnetworks had lower values of nodes, edges and clustering coefficient and higher values of average path length and modularity in all three microhabitats (Table S5), suggesting that microbial co-occurrence networks were more complex in the high-latitude areas than in the low-latitude areas. This result is consistent with the findings of Gao et al. (46), who found that the network complexity of soil bacterial communities increased with increasing latitude in Chinese coastal wetlands. One explanation for such latitudinal patterns of microbial network complexity is the great variations in water-energy conditions between the low-latitude and high-latitude areas in China (8). The low precipitation in high-latitude areas may lead to weak niche differentiation, which enhances species competition for limited resources and causes strong interactions between soil microorganisms.

### Contributions of environmental and microbial factors to the EMF

The contributions of abiotic and biotic factors to EMF have been investigated in drylands, forests, grasslands, and agricultural ecosystems (2, 14, 47). In line with most previous studies, we found that abiotic factors, including geographic location, climate, and edaphic properties, explained up to 52.66% and 55.97% of the variance in EMF_average_ and EMF_weighted_, respectively (Fig. 6). This result suggests that environmental conditions rather than microbial diversity and network complexity drive EMF in rivers along a latitudinal gradient across eastern China. Besides geographic and climatic factors, local soil physicochemical properties (e.g., pH and texture) also played a vital role in influencing the EMF (Fig. 6; Fig. S9). Xiong et al. (48) revealed that an elevated soil pH could inhibit the activity and function of microbial communities by altering the homeostasis of microbial cells and reducing the availability of soil nutrients. In addition, several studies have shown that soil texture is an important driver of several ecological functions, including but not limited to carbon sequestration, nutrient retention, and water infiltration (49, 50). Compared with coarse and sandy soils, soils with high clay and silt contents have higher water-holding capacity and nutrient availability, which can increase ecosystem productivity and multifunctionality. Therefore, EMF has been frequently reported to be positively related to silt and clay contents but negatively correlated with sand content in soils (7, 51).

Microorganisms are critical drivers of numerous ecosystem functions and multifunctionality (15), although microbial diversity and network complexity contributed little to explaining EMF in rivers across eastern China (Fig. 6 and 7). The importance of microbial diversity in determining the EMF has been documented in a wide range of terrestrial ecosystems (14, 47, 52). For example, Jing et al. (12) reported a positive relationship between bacterial species diversity and EMF in grasslands of the Tibetan Plateau, China. Higher diversity in the microbial communities implies a higher functional uniqueness, which may enhance biogeochemical process rates, promote plant growth, and reduce nutrient leaching loss (26). Relatively few studies have focused on the influences of microbial network complexity on EMF (16, 26, 38). In the present study, we found that one network complexity parameter (i.e., betweenness centralization) could explain the variance in EMF_average_ (Fig. 6A). Similarly, Jiao et al. (14) reported that node and edge number of microbial networks were positively correlated with EMF, and average path length was negatively related to EMF. These findings imply that microbial network complexity is important in shaping ecosystem functions, but a higher microbial network complexity may not necessarily mean a higher EMF.

### Conclusions

This study indicated that EMF significantly decreased with increasing latitude in channel sediments and riparian soils of rivers across eastern China. Such latitudinal patterns of EMF were likely a result of the collective effects of complex interactions of climate, soil physicochemical properties, and microbial communities that change in relation to the latitude. Specifically, environmental conditions, especially geographic and climatic factors, were the main drivers of EMF. Microbial diversity and network complexity contributed little to explaining EMF, and only betweenness centralization had a significant relationship with EMF. Overall, this study fills a critical knowledge gap regarding the latitudinal patterns and drivers of EMF in river ecosystems and gives new insights into how microbial diversity and network complexity affect EMF from a metagenomic perspective.

## Data Availability

All raw sequences were deposited in the National Center for Biotechnology Information (NCBI) Sequence Read Archive (SRA) database with the accession number PRJNA779832. The data that support the findings of this study are available from Figshare at https://doi.org/10.6084/m9.figshare.23702667.v2

## SUPPLEMENTAL MATERIAL

Supplemental material msystems.00147-24-s0001.docx Supplemental figures and tables.

ASM does not own the copyrights to Supplemental Material that may be linked to, or accessed through, an article. The authors have granted ASM a non-exclusive, world-wide license to publish the Supplemental Material files. Please contact the corresponding author directly for reuse.
