# Supplementary material for: Linking ecosystem multifunctionality to microbial community features in rivers along a latitudinal gradient
Source: mSystems. 2024 Mar 6;9(4):e00147-24. doi: 10.1128/msystems.00147-24 (PMC11019869; doi:10.1128/msystems.00147-24)
Supplement: Supplemental material — Supplemental figures and tables. [file msystems.00147-24-s0001.docx]

**Supporting Information**

**Linking ecosystem multifunctionality to microbial community features in rivers along a latitudinal gradient**

Miaomiao Cai ^a, b^, Caifang Zhang ^a, c^, Caroline Njambi Ndungu ^a, c^, Guihua Liu ^a, b^, Wenzhi Liu ^a, b#^, Quanfa Zhang ^a, b^

*^a^CAS Key Laboratory of Aquatic Botany and Watershed Ecology, Wuhan Botanical Garden, Chinese Academy of Sciences, Wuhan, 430074, China*

*^b^Danjiangkou Wetland Ecosystem Field Scientific Observation and Research Station, Chinese Academy of Sciences & Hubei Province, Wuhan, 430074, China*

*^c^University of Chinese Academy of Sciences, Beijing, 100049, China*

The first two authors contributed equally to this work.

**#Corresponding author**

*Phone: +86 27 87700849*

*Fax: +86 27 87700877*

*Email:* [*liuwz@wbgcas.cn*](mailto:liuwz@wbgcas.cn)

**Running head:** Latitudinal patterns of multifunctionality

# Information for supplemental materials

**Fig. S1** Sampling sites across eastern China. The blue line is the Qinling–Huaihe Line, which separates the 30 sampling sites into low-latitude (orange dots) and high-latitude (green dots) groups.

**Fig. S2** Individual ecosystem functions used to calculate multifunctionality index. The functions within the same functional group (i.e., nitrogen cycling, nutrient pools, plant productivity, and water quality) were weighted equally to avoid overweighting of certain aspects of ecosystem functioning and assessment bias. All abbreviations are explained in Table S2.

**Fig. S3** The difference in weighted multifunctionality index (EMF_weighted_) in rhizosphere soil, bulk soil, and sediment between the high-latitude and low-latitude groups. * indicates *p* < 0.05.

**Fig. S4** Spearman correlation coefficients between latitude and individual ecosystem functions and multifunctionality (EMF_average_ and EMF_weighted_). Edge width corresponds to the coefficients. Green lines indicate positive effects, while orange lines indicate negative effects. * and *** indicate *p* < 0.05 and 0.001, respectively.

**Fig. S5** Liner regression model describing the relationship between latitude and weighted multifunctionality index (EMF_weighted_) among habitats (A: Rhizosphere soil; B: Bulk soil; C: Sediment; D: All samples).

**Fig. S6** The relationship between latitude and the number of functions exceeding a threshold level from 5% and 95% of maximum functioning (i.e., multi-threshold multifunctionality index) in rhizosphere soil (A), bulk soil (B), and sediment (C). Effects of latitude on multiple-threshold multifunctionality index in rhizosphere soil (D), bulk soil (E), and sediment (F). The black points and shadowed area indicate the slope and the 95% confidence interval of the regressions.

**Fig. S7** Distance-based redundancy analysis (dbRDA) of microbial community (A: species level) and functional genes (B: KEGG Orthology) based on Bray-Curtis dissimilarities in three habitats along a latitudinal gradient.

**Fig. S8** The correlation between latitude and microbial diversity, microbial network complexity, edaphic factors, and climatic factors based on Spearman correlation analyses. *, **, and *** indicate *p* < 0.05, 0.01, and 0.001, respectively.

**Fig. S9** The correlation between predictive factors and individual ecosystem functions and multifunctionality based on Spearman correlation analyses. * and ** indicate *p* < 0.05 and 0.01, respectively.

**Fig. S10** The correlation between predictive factors and individual ecosystem functions and multifunctionality in rhizosphere soil based on Spearman correlation analyses. * and ** indicate *p* < 0.05 and 0.01, respectively.

**Fig. S11** The correlation between predictive factors and individual ecosystem functions and multifunctionality in bulk soil based on Spearman correlation analyses. * and ** indicate *p* < 0.05 and 0.01, respectively.

**Fig. S12** The correlation between predictive factors and individual ecosystem functions and multifunctionality in sediment based on Spearman correlation analyses. * and ** indicate *p* < 0.05 and 0.01, respectively.

**Table S1** Geographic location and climatic features of the sampling sites.

**Table S2** The abbreviations of the microbial diversity and ecosystem function parameters.

**Table S3** Mann-Whitney U test of 18 single functions between low-latitude and high-latitude group.

**Table S4** α diversity of the microbial community (species level) and functional genes (KEGG Orthology).

**Table S5** Topological features of microbial co-occurrence networks in rhizosphere soil, bulk soil, and sediment.

**Table S6** Mann-Whitney U test of subnetwork topological features between low-latitude and high-latitude group.


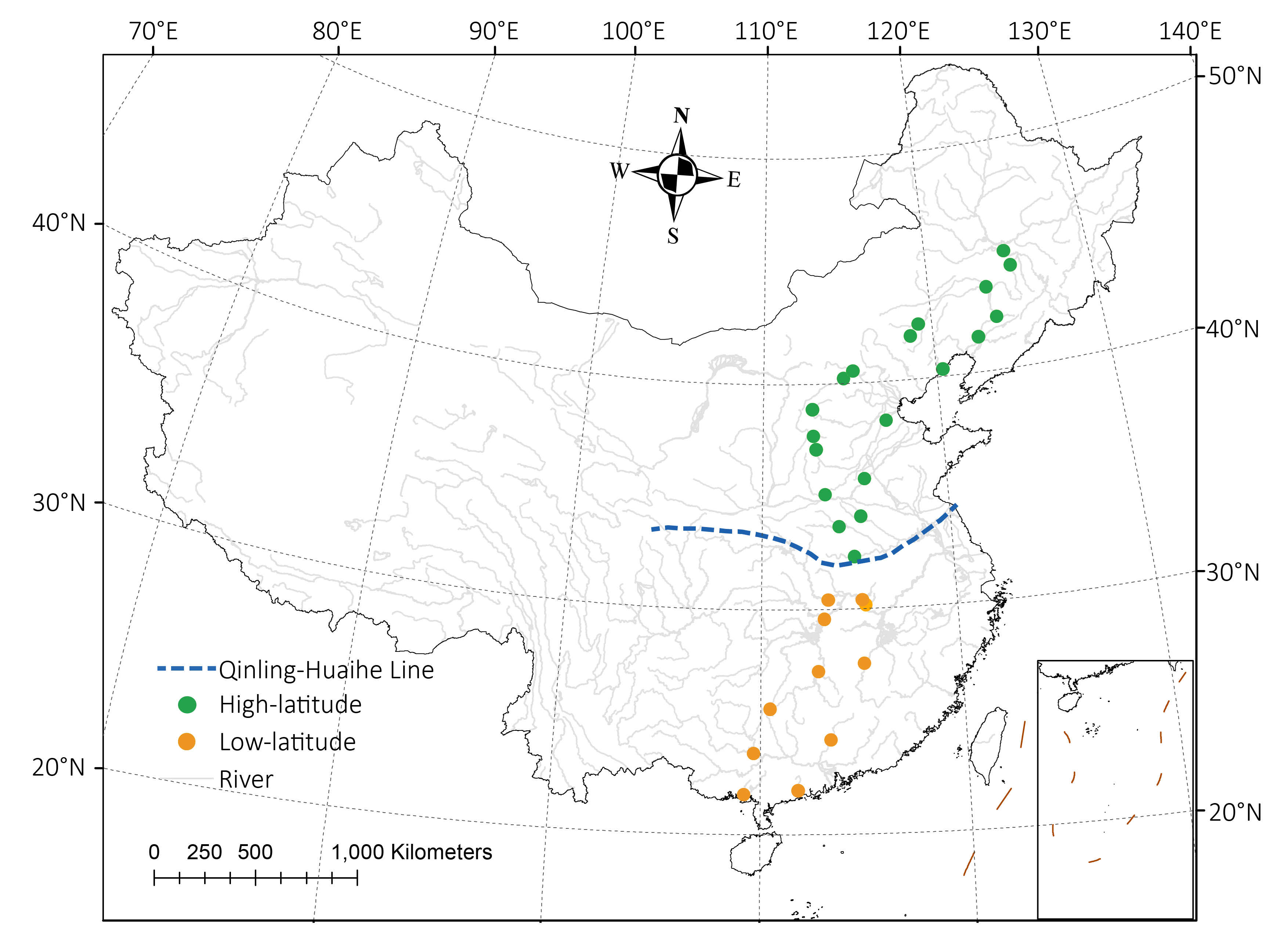


**Fig. S1** Sampling sites across eastern China. The blue line is the Qinling–Huaihe Line, which separates the 30 sampling sites into low-latitude (orange dots) and high-latitude (green dots) groups.


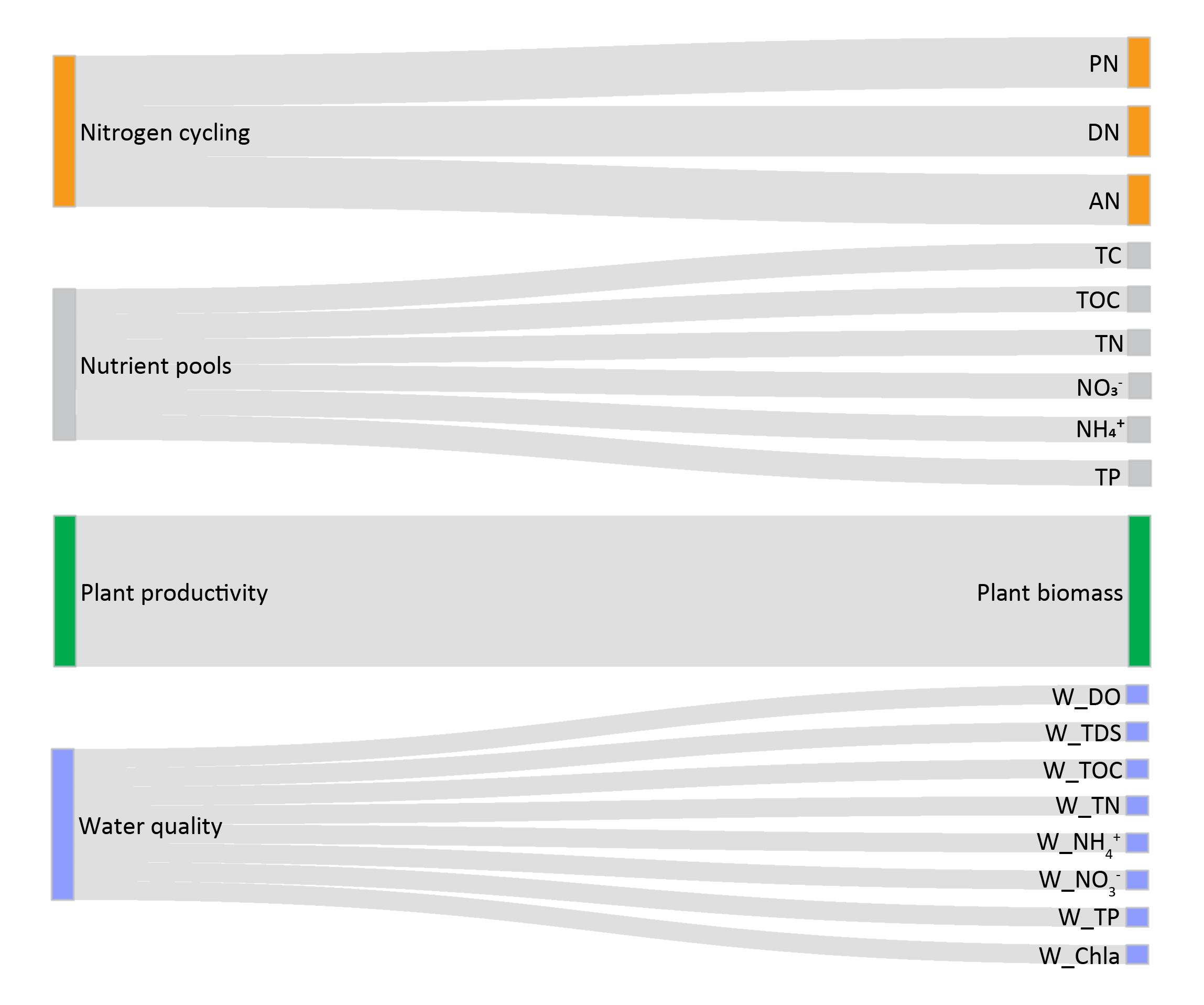


**Fig. S2** Individual ecosystem functions used to calculate multifunctionality index. The functions within the same functional group (i.e., nitrogen cycling, nutrient pools, plant productivity, and water quality) were weighted equally to avoid overweighting of certain aspects of ecosystem functioning and assessment bias. All abbreviations are explained in Table S2.


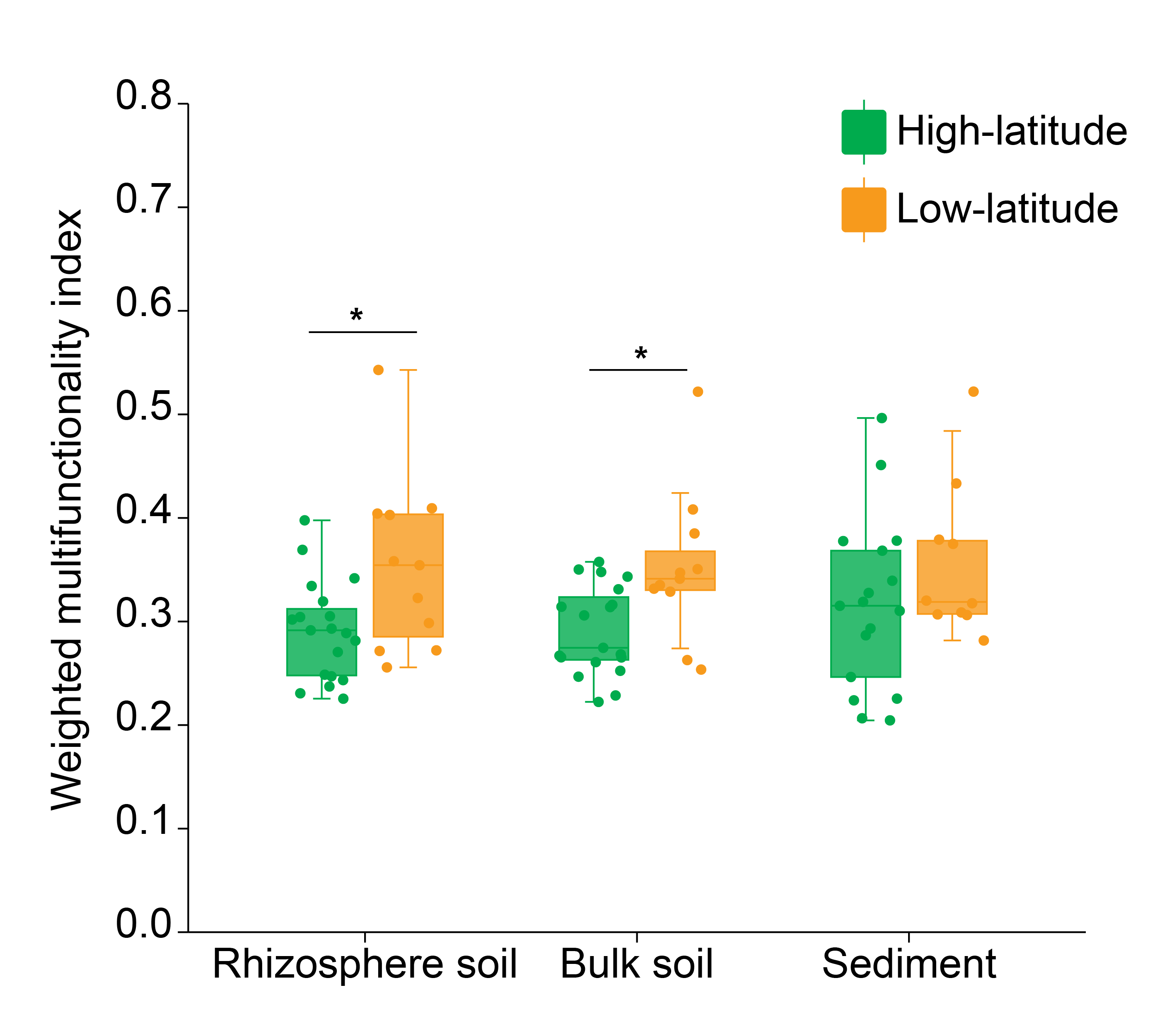


**Fig. S3** The difference in weighted multifunctionality index (EMF_weighted_) in rhizosphere soil, bulk soil, and sediment between the high-latitude and low-latitude groups. * indicate *p* < 0.05.


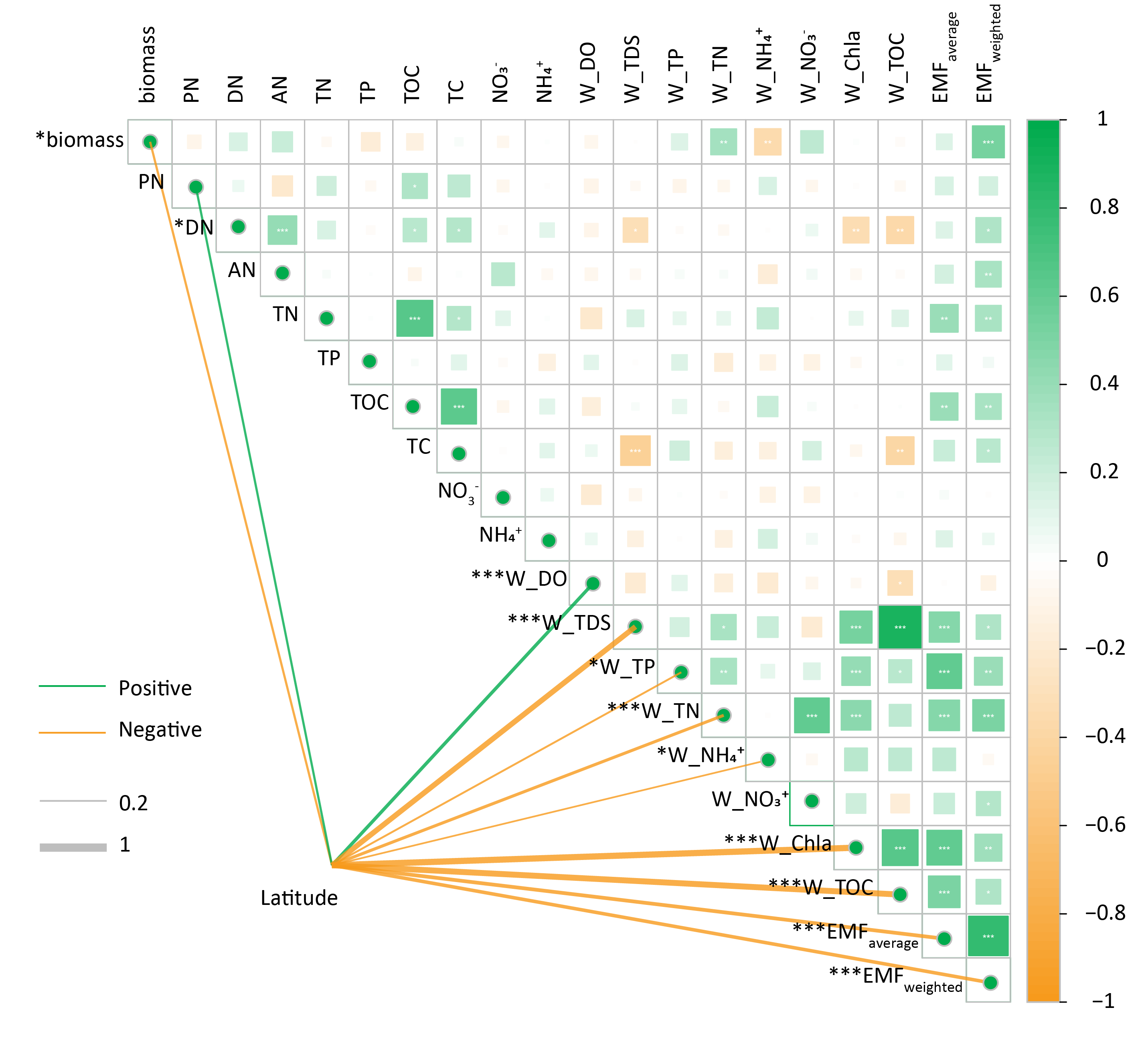


**Fig. S4** Spearman correlation coefficients between latitude and individual ecosystem functions and multifunctionality (EMF_average_ and EMF_weighted_). Edge width corresponds to the coefficients. Green lines indicate positive effects, while orange lines indicate negative effects. * and *** indicate *p* < 0.05 and 0.001, respectively.


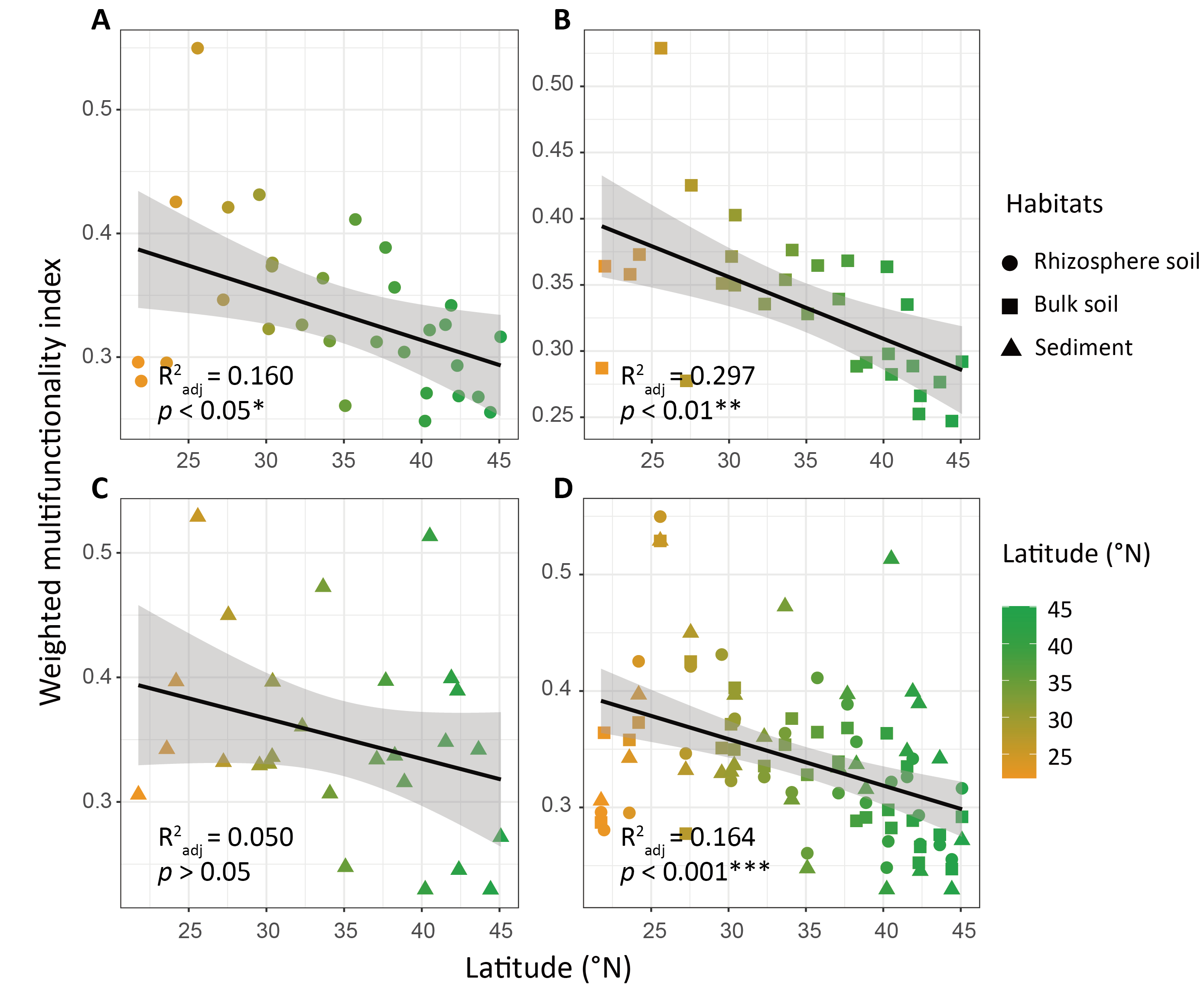


**Fig. S5** Liner regression model describing the relationship between latitude and weighted multifunctionality index (EMF_weighted_) among habitats (A: Rhizosphere soil; B: Bulk soil; C: Sediment; D: All samples).


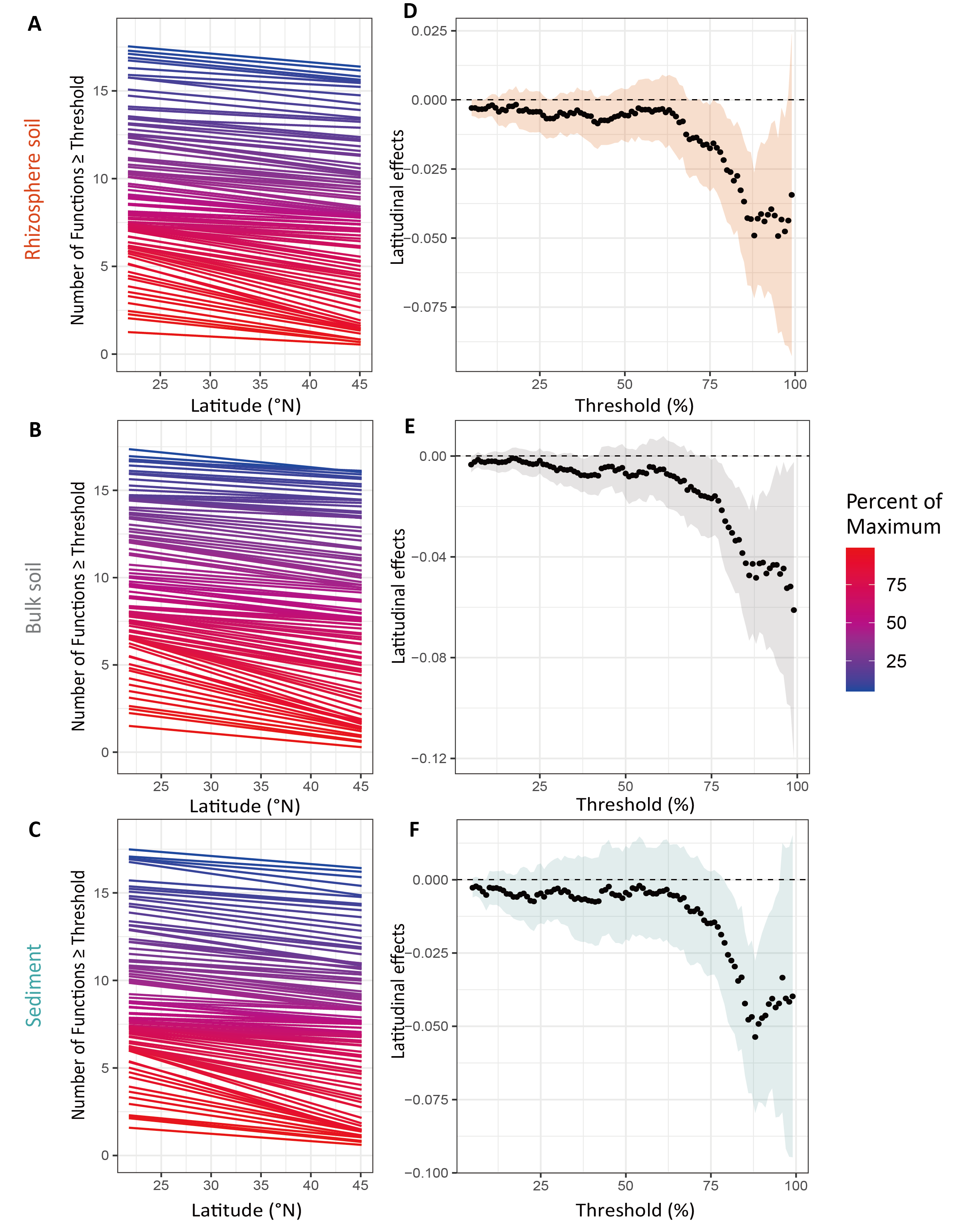


**Fig. S6** The relationship between latitude and the number of functions exceeding a threshold level from 5% and 95% of maximum functioning (i.e., multi-threshold multifunctionality index) in rhizosphere soil (A), bulk soil (B), and sediment (C). Effects of latitude on multiple-threshold multifunctionality index in rhizosphere soil (D), bulk soil (E), and sediment (F). The black points and shadowed area indicate the slope and the 95% confidence interval of the regressions.


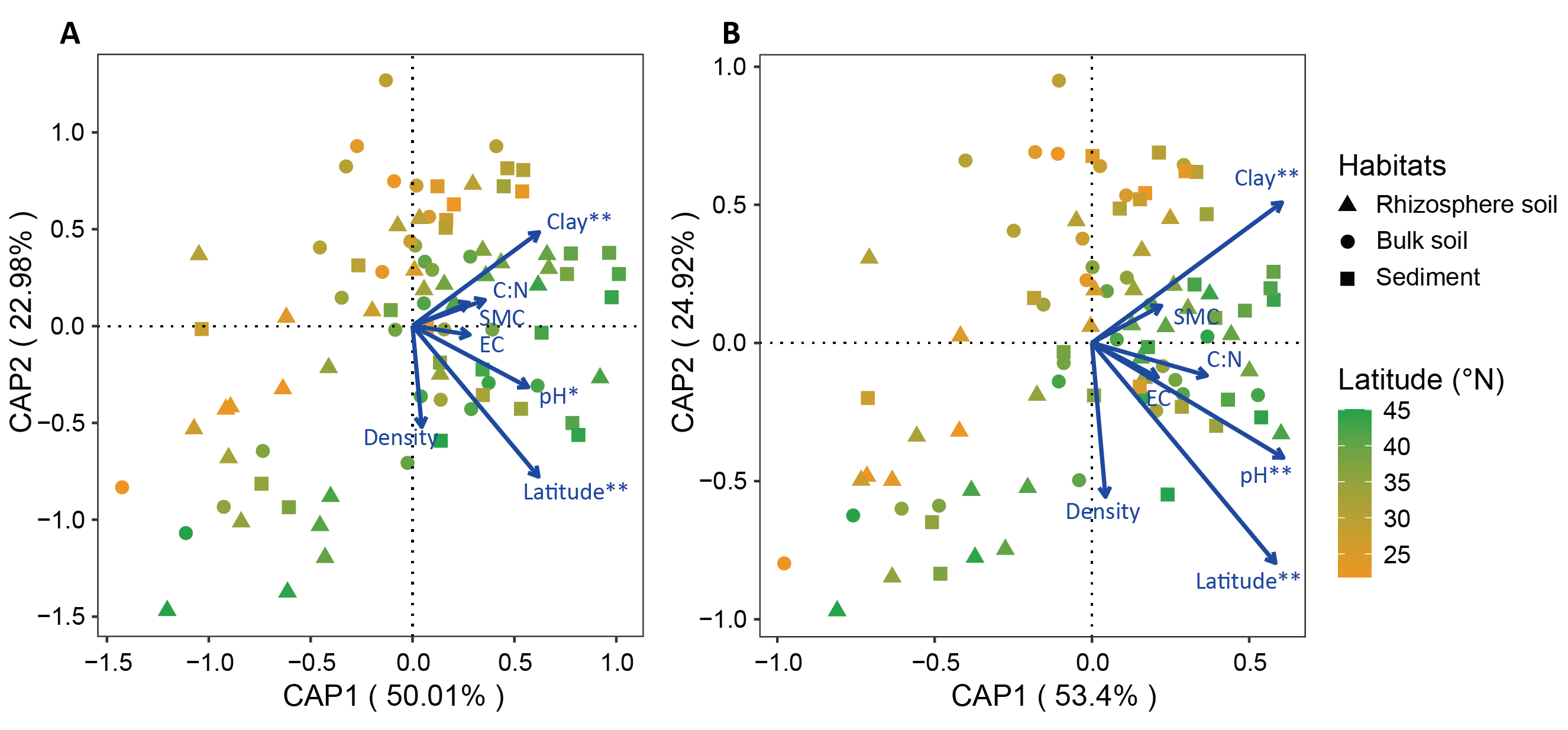


**Fig. S7** Distance-based redundancy analysis (dbRDA) of microbial community (A: species level) and functional genes (B: KEGG Orthology) based on Bray-Curtis dissimilarities in three habitats along a latitudinal gradient.


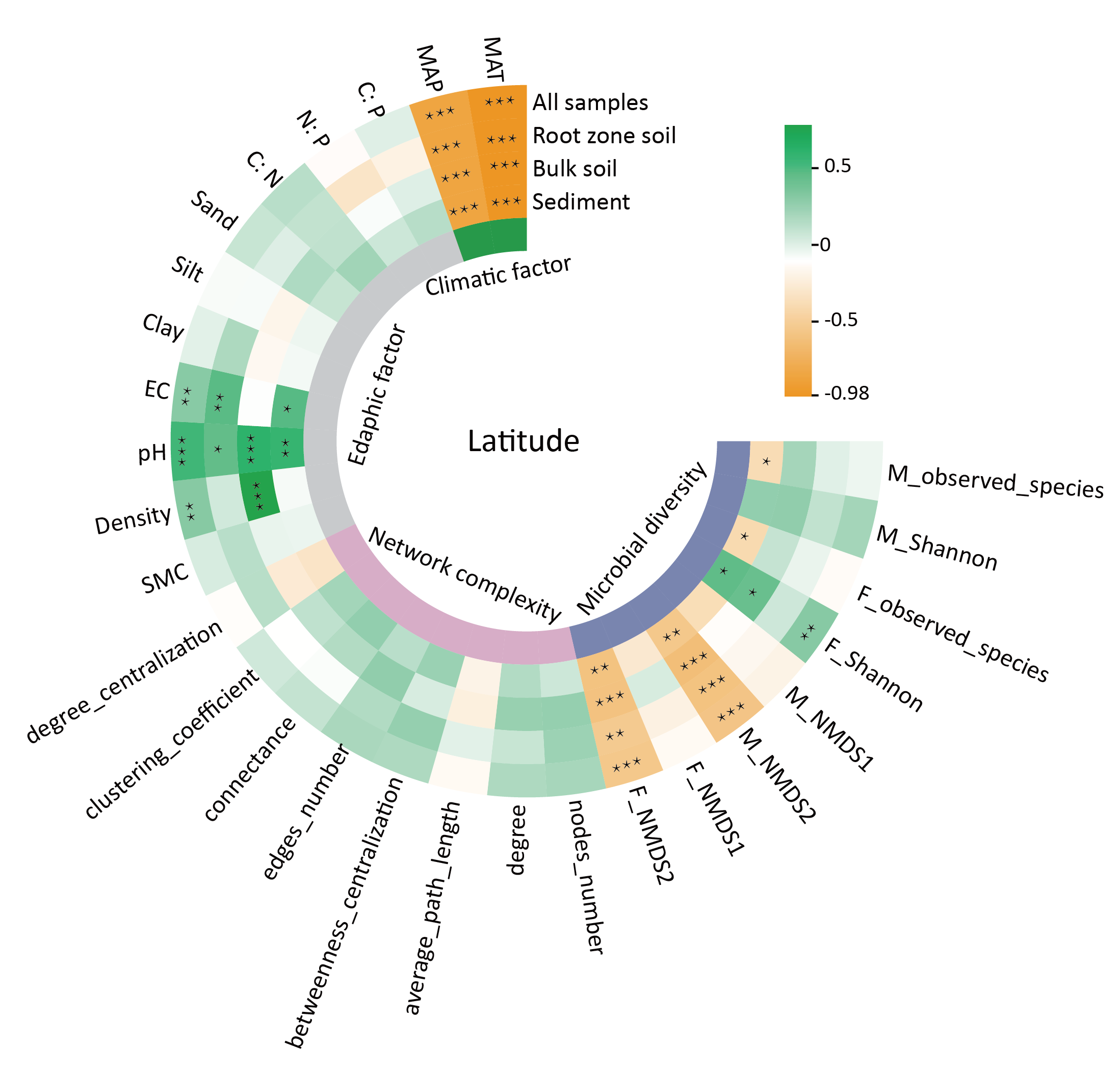


**Fig. S8** The correlation between latitude and microbial diversity, microbial network complexity, edaphic factors, and climatic factors based on Spearman correlation analyses. *, **, and *** indicate *p* < 0.05, 0.01, and 0.001, respectively.


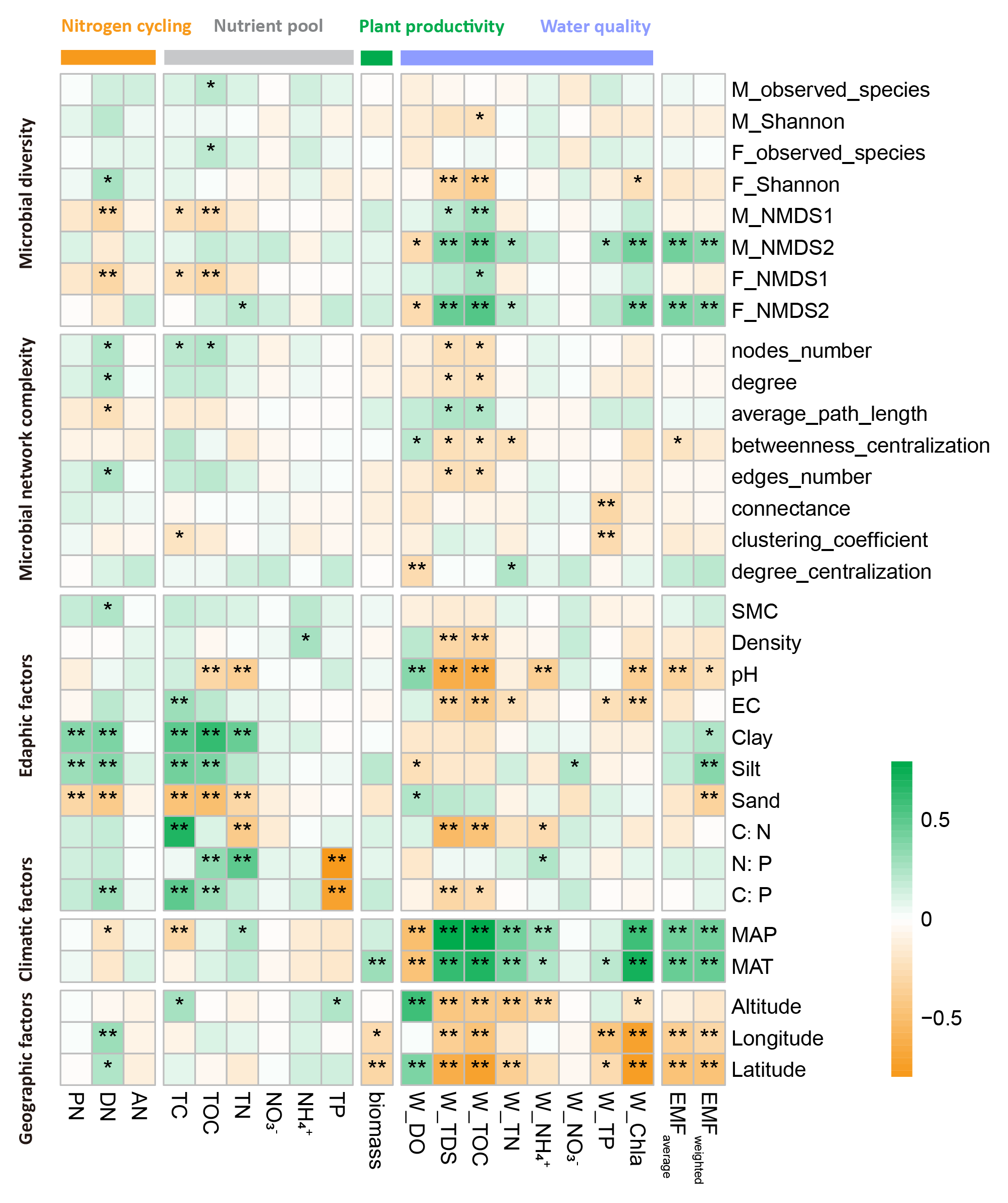


**Fig. S9** The correlation between predictive factors and individual ecosystem functions and multifunctionality based on Spearman correlation analyses. * and ** indicate *p* < 0.05 and 0.01, respectively.


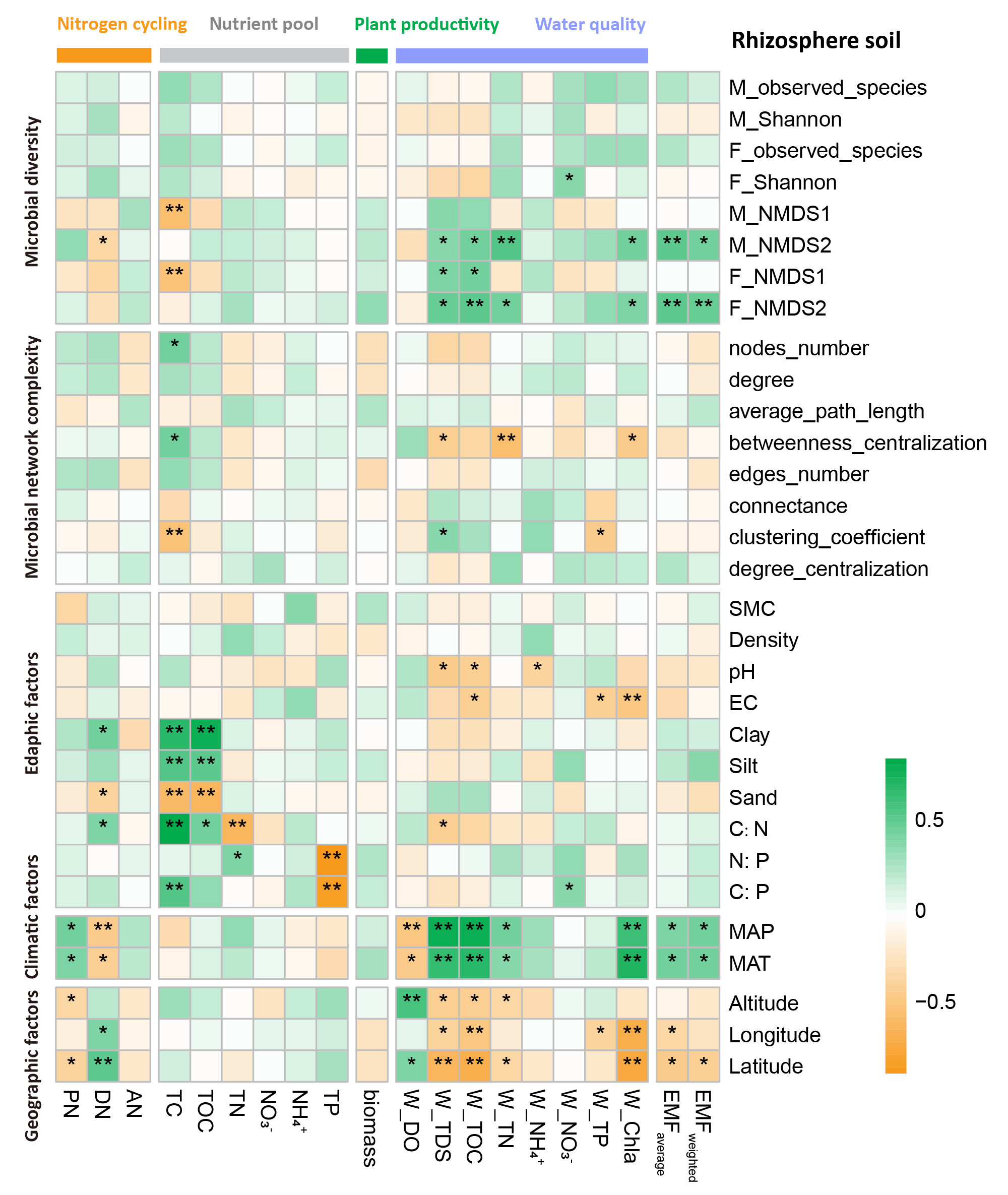


**Fig. S10** The correlation between predictive factors and individual ecosystem functions and multifunctionality in rhizosphere soil based on Spearman correlation analyses. * and ** indicate *p* < 0.05 and 0.01, respectively.


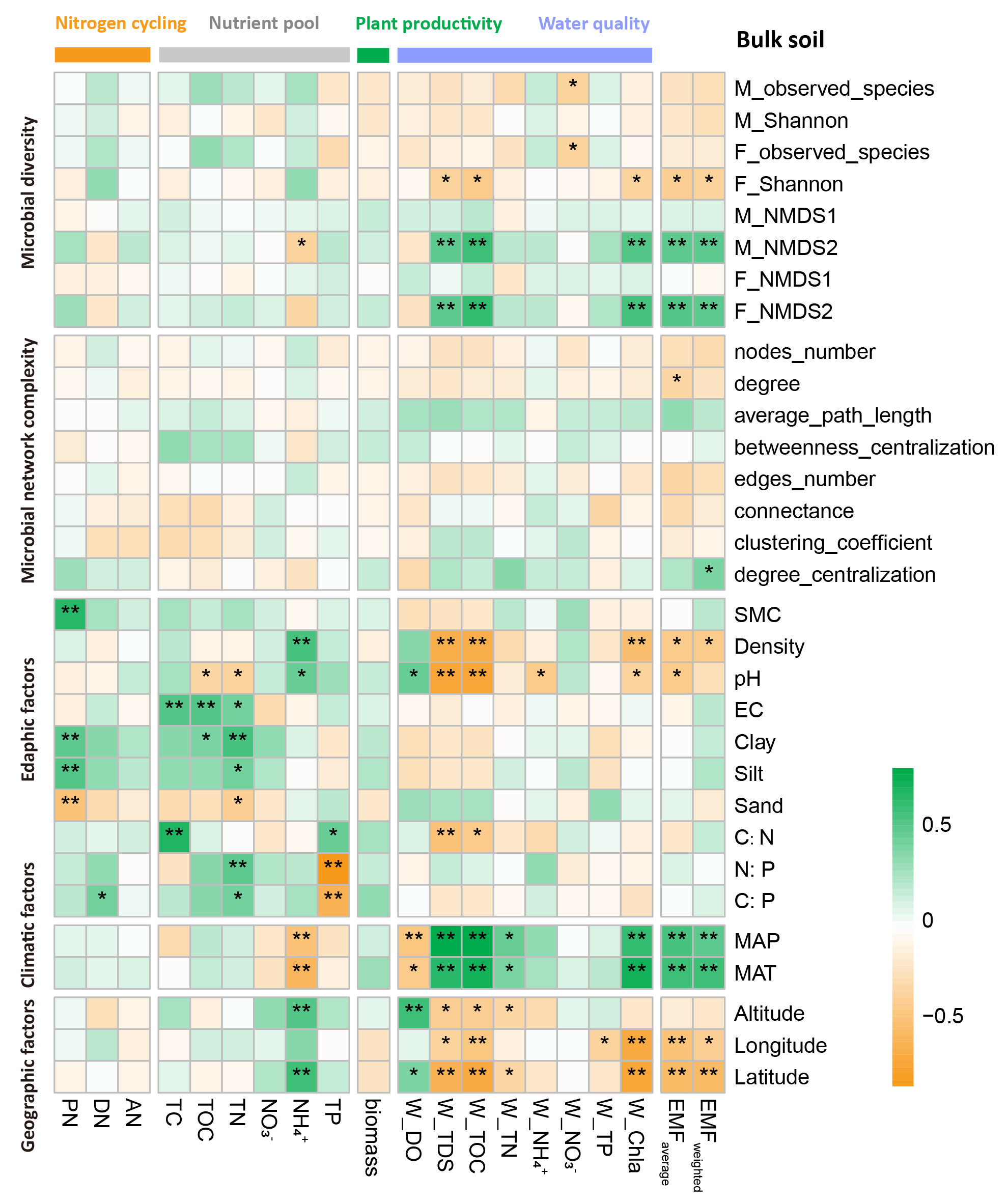


**Fig. S11** The correlation between predictive factors and individual ecosystem functions and multifunctionality in bulk soil based on Spearman correlation analyses. * and ** indicate *p* < 0.05 and 0.01, respectively.


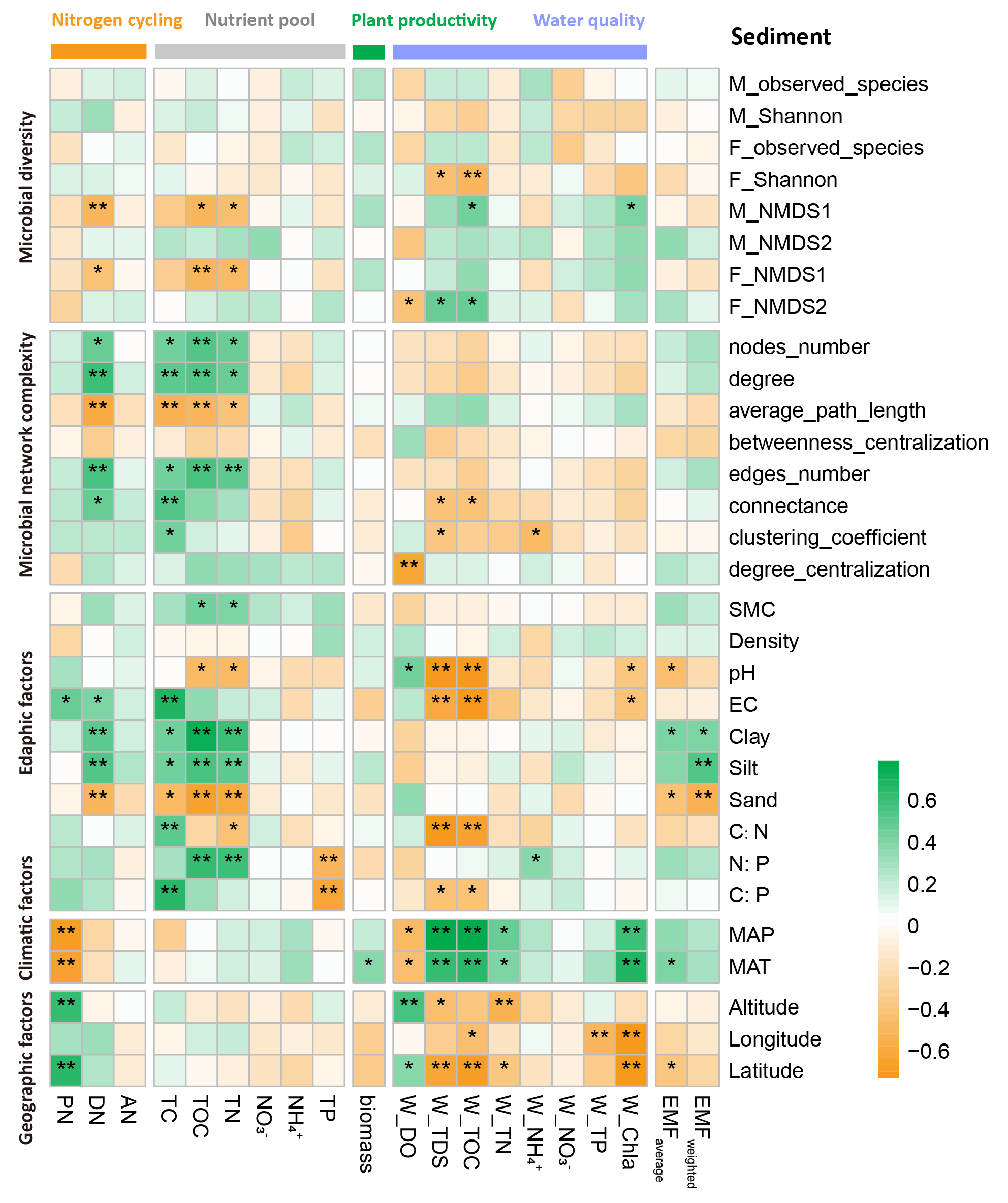


**Fig. S12** The correlation between predictive factors and individual ecosystem functions and multifunctionality in sediment based on Spearman correlation analyses. * and ** indicate *p* < 0.05 and 0.01, respectively.

**Table S1** Geographic location and climatic features of the sampling sites.

| Sites | Description | Latitude  ( ̊ N) | Longitude  ( ̊ E) | Altitude  (m) | MAP  (mm) | MAT  (℃) | Group |
| --- | --- | --- | --- | --- | --- | --- | --- |
| 1 | Huaihe river | 32.305 | 114.852 | 31.9 | 1032 | 15.567 | High-latitude |
| 2 | Huiji River | 34.085 | 115.256 | 41.2 | 733 | 14.608 | High-latitude |
| 3 | Yellow River | 35.736 | 115.556 | 46.6 | 589 | 13.808 | High-latitude |
| 4 | Jiedijian river | 38.261 | 116.930 | 1.1 | 493 | 13.092 | High-latitude |
| 5 | Liugu river | 40.321 | 120.378 | 0.2 | 582 | 9.275 | High-latitude |
| 6 | Liaohe river | 41.536 | 122.655 | 11.3 | 612 | 8.558 | High-latitude |
| 7 | Chaihe river | 42.294 | 123.872 | 63 | 654 | 7.800 | High-latitude |
| 8 | Yitong river | 44.425 | 125.196 | 164.1 | 538 | 5.275 | High-latitude |
| 9 | Songhua river | 45.085 | 124.922 | 133.4 | 475 | 4.700 | High-latitude |
| 10 | Xinkai river | 43.650 | 123.529 | 118.7 | 465 | 7.033 | High-latitude |
| 11 | Yingjin river | 42.385 | 119.212 | 498.9 | 366 | 7.608 | High-latitude |
| 12 | Xibo river | 41.902 | 118.688 | 770.6 | 412 | 7.083 | High-latitude |
| 13 | Yanghe river | 40.516 | 115.170 | 562.2 | 380 | 9.608 | High-latitude |
| 14 | Sanggan river | 40.221 | 114.620 | 806.7 | 375 | 7.925 | High-latitude |
| 15 | Hutuo river | 38.879 | 112.780 | 803.5 | 409 | 9.108 | High-latitude |
| 16 | Xiaohe river | 37.680 | 112.823 | 800.9 | 413 | 9.850 | High-latitude |
| 17 | Zhuozhang river | 37.104 | 112.944 | 1000.2 | 552 | 9.350 | High-latitude |
| 18 | Qinhe river | 35.087 | 113.388 | 88.7 | 579 | 14.467 | High-latitude |
| 19 | Shahe river | 33.650 | 114.098 | 47.8 | 771 | 15.050 | High-latitude |
| 20 | Hanjiang river | 30.397 | 113.427 | 2.5 | 1178 | 16.983 | Low-latitude |
| 21 | Yangtze river | 29.558 | 113.215 | 23.2 | 1256 | 17.192 | Low-latitude |
| 22 | Xiangjiang river | 27.234 | 112.877 | 34.7 | 1432 | 18.042 | Low-latitude |
| 23 | Lijiang river | 25.585 | 110.466 | 182.5 | 1662 | 18.492 | Low-latitude |
| 24 | Qianjiang river | 23.589 | 109.68 | 38.1 | 1284 | 21.600 | Low-latitude |
| 25 | Nanliu river | 21.762 | 109.234 | 3.9 | 1664 | 22.442 | Low-latitude |
| 26 | Moyang river | 21.956 | 111.809 | -1.4 | 2152 | 23.025 | Low-latitude |
| 27 | Beijiang river | 24.189 | 113.426 | 22.08 | 1719 | 21.333 | Low-latitude |
| 28 | Ganjiang river | 27.551 | 115.162 | 26.45 | 1553 | 18.050 | Low-latitude |
| 29 | Qishui river | 30.160 | 115.345 | 7 | 1384 | 17.283 | Low-latitude |
| 30 | Xishui river | 30.368 | 115.168 | 4.5 | 1362 | 17.117 | Low-latitude |

**Table S2** The abbreviations of the microbial diversity and ecosystem function parameters.

| Variables | Abbreviation |
| --- | --- |
| Observed species number of microbial community | M_observed_species |
| Shannon index of microbial community | M_Shannon |
| Observed species number of functional genes | F_observed_species |
| Shannon index of functional genes | F_Shannon |
| Axis 1 of non-metric multidimensional scaling of microbial community | M_NMDS1 |
| Axis 2 of non-metric multidimensional scaling of microbial community | M_NMDS2 |
| Axis 1 of non-metric multidimensional scaling of functional genes | F_NMDS1 |
| Axis 2 of non-metric multidimensional scaling of functional genes | F_NMDS2 |
| Soil/sediment moisture content | SMC |
| Density of soil/sediment | Density |
| Electronic conductivity of soil/sediment | EC |
| The ratio of total carbon to total nitrogen | C: N |
| The ratio of total nitrogen to total phosphorus | N: P |
| The ratio of total carbon to total phosphorus | C: P |
| Potential nitrification rate of soil/sediment | PN |
| Denitrification rate of soil/sediment | DN |
| Anammox rate of soil/sediment | AN |
| Total carbon content of soil/sediment | TC |
| Total organic carbon content of soil/sediment | TOC |
| Total nitrogen content of soil/sediment | TN |
| Nitrate content of soil/sediment | NO_3_^-^ |
| Ammonium content of soil/sediment | NH_4_^+^ |
| Total phosphorus content of soil/sediment | TP |
| Dissolved oxygen of water | W_DO |
| Total dissolved solids of water | W_TDS |
| Total organic carbon of water | W_TOC |
| Total nitrogen content of water | W_TN |
| Ammonium content of water | W_NH_4_^+^ |
| Nitrate content of water | W_NO_3_^-^ |
| Total phosphorus content of water | W_TP |
| Chlorophyll a of water | W_Chla |

**Table S3** Mann-Whitney U test of 18 single functions between low-latitude and high-latitude group.

| Functions | Habitat types | | | | | | | | | | |
| --- | --- | --- | --- | --- | --- | --- | --- | --- | --- | --- | --- |
|  | Rhizosphere soil | | | | Bulk soil | | | | Sediment | | |
|  | Mean | | *p* | | Mean | | *p* | | Mean | | *p* |
|  | High-latitude | Low-latitude |  | High-latitude | | Low-latitude |  | High-latitude | | Low-latitude |  |
| Biomass (g) | 256.19 | 400.51 | 0.395 | 256.19 | | 400.51 | 0.395 | 228.59 | | 426.90 | 0.155 |
| PN (mg N/kg·d) | 9.28 | 49.71 | 0.001** | 5.17 | | 22.63 | 0.35 | 19.75 | | 3.97 | 0.001  ** |
| DN (ngN/g·h) | 610.89 | 239.85 | 0.001** | 493.89 | | 433.38 | 0.981 | 819.84 | | 354.75 | 0.155 |
| AN (ngN/g·h) | 222.34 | 233.56 | 0.767 | 544.10 | | 468.47 | 0.827 | 426.33 | | 376.84 | 0.675 |
| TC (mg/g) | 14.64 | 9.80 | 0.134 | 11.71 | | 9.49 | 0.232 | 21.42 | | 10.02 | 0.264 |
| TOC (mg/g) | 7.36 | 5.46 | 0.8 | 4.24 | | 4.31 | 0.668 | 12.80 | | 5.01 | 0.902 |
| TN (mg/kg) | 635.06 | 891.88 | 0.052 | 748.31 | | 882.28 | 0.372 | 1283.67 | | 1077.88 | 0.473 |
| NO_3_^-^ (mg/kg) | 0.51 | 0.47 | 0.832 | 0.94 | | 0.60 | 0.709 | 2.62 | | 1.48 | 0.643 |
| NH_4_^+^ (mg/kg) | 14.16 | 8.48 | 0.307 | 10.14 | | 3.17 | 0*** | 7.36 | | 12.14 | 0.127 |
| TP (mg/kg) | 22.83 | 13.66 | 0.933 | 13.63 | | 12.34 | 0.525 | 14.49 | | 13.94 | 0.786 |
| W_DO (mg/L) | 6.49 | 4.18 | 0.016* | 6.49 | | 4.18 | 0.016* | 6.59 | | 4.21 | 0.027  * |
| W_TDS (mg/L) | 501.75 | 149.23 | 0*** | 501.75 | | 149.23 | 0*** | 505.72 | | 156.35 | 0*** |
| W_TOC (mg/L) | 34.40 | 13.78 | 0*** | 34.40 | | 13.78 | 0*** | 35.27 | | 14.29 | 0*** |
| W_TN (mg/L) | 4.66 | 2.49 | 0.042* | 4.66 | | 2.49 | 0.042* | 4.77 | | 2.33 | 0.04* |
| W_NH_4_^+^ (mg/L) | 0.39 | 0.36 | 0.25 | 0.39 | | 0.36 | 0.25 | 0.39 | | 0.38 | 0.443 |
| W_NO_3_^-^ (mg/L) | 1.81 | 1.77 | 0.395 | 1.81 | | 1.77 | 0.395 | 1.82 | | 1.68 | 0.505 |
| W_TP (mg/L) | 0.04 | 0.02 | 0.471 | 0.04 | | 0.02 | 0.471 | 0.03 | | 0.02 | 0.309 |
| W_Chla (μg/L) | 21.84 | 8.35 | 0.005** | 21.84 | | 8.35 | 0.005  ** | 23.37 | | 9.17 | 0.009  ** |

Notes: *, ** and *** indicate *p* < 0.05, *p* < 0.01 and 0.001, respectively

**Table S4** α diversity of the microbial community (species level) and functional genes (KEGG Orthology).

| Functions | Habitat types | | | | | | | | | | |
| --- | --- | --- | --- | --- | --- | --- | --- | --- | --- | --- | --- |
|  | Rhizosphere soil | | | | Bulk soil | | | | Sediment | | |
|  | Mean | | *p* | | Mean | | *p* | | Mean | | *p* |
|  | High-latitude | Low-latitude |  | High-latitude | | Low-latitude |  | High-latitude | | Low-latitude |  |
| M_observed_species | 24938 | 25015 | 1 | 25168 | | 25042 | 0.703 | 25106 | | 25939 | 0.023* |
| M_Shannon | 6.8133 | 6.7799 | 0.767 | 6.8505 | | 6.7872 | 0.641 | 6.9255 | | 6.9141 | 0.551 |
| F_observed_species | 8029 | 8036 | 0.832 | 8010 | | 8032 | 0.899 | 7997.5 | | 8284 | 0.014* |
| F_Shannon | 11.033 | 11.014 | 0.525 | 11.0347 | | 10.9949 | 0.037* | 11.0676 | | 11.0378 | 0.014* |

**Table S5** Topological features of microbial co-occurrence networks in rhizosphere soil, bulk soil, and sediment.

| Habitat types | Groups | Nodes | Edges | Average path length | Clustering coefficient | Modularity |
| --- | --- | --- | --- | --- | --- | --- |
| Rhizosphere soil | High-latitude | 3213 | 429329 | 2.9871 | 0.6350 | 0.206 |
|  | Low-latitude | 2159 | 24565 | 4.6984 | 0.4663 | 0.604 |
| Bulk soil | High-latitude | 2884 | 115449 | 3.5195 | 0.5478 | 0.499 |
|  | Low-latitude | 1487 | 4607 | 7.6926 | 0.4775 | 0.768 |
| Sediment | High-latitude | 2775 | 61659 | 4.0226 | 0.5967 | 0.669 |
|  | Low-latitude | 1620 | 5149 | 6.5172 | 0.4769 | 0.780 |

**Table S6** Mann-Whitney U test of subnetwork topological features between low-latitude and high-latitude group.

| Topological features | Habitat types | | | | | | | | | | |
| --- | --- | --- | --- | --- | --- | --- | --- | --- | --- | --- | --- |
|  | Rhizosphere soil | | | | Bulk soil | | | | Sediment | | |
|  | Mean | | *p* | | Mean | | *p* | | Mean | | *p* |
|  | High-latitude | Low-latitude |  | High-latitude | | Low-latitude |  | High-latitude | | Low-latitude |  |
| nodes_  number | 349.32 | 339.45 | 0.525 | 349.32 | | 339.45 | 0.2 | 362.82 | | 357.90 | 0.711 |
| degree | 64.93 | 64.01 | 0.966 | 64.93 | | 64.01 | 0.216 | 69.50 | | 68.51 | 0.902 |
| average_path_  length | 1.94 | 1.95 | 0.899 | 1.94 | | 1.95 | 0.232 | 1.93 | | 1.93 | 0.941 |
| betweenness_  centralization | 0.02 | 0.02 | 0.25 | 0.02 | | 0.02 | 0.395 | 0.02 | | 0.02 | 0.786 |
| edges_  number | 11370 | 10932 | 0.735 | 11370 | | 10932 | 0.216 | 12708 | | 12370 | 0.941 |
| connectance | 0.19 | 0.19 | 0.35 | 0.19 | | 0.19 | 0.328 | 0.19 | | 0.19 | 0.863 |
| diameter | 4.53 | 4.36 | 0.171 | 4.53 | | 4.36 | 0.471 | 4.71 | | 4.70 | 0.98 |
| edge_  connectivity | 0.32 | 0.55 | 0.8 | 0.32 | | 0.55 | 0.641 | 0.47 | | 0.50 | 0.863 |
| clustering_  coefficient | 0.36 | 0.38 | 0.307 | 0.36 | | 0.38 | 0.011* | 0.37 | | 0.36 | 0.537 |
| degree_  centralization | 0.36 | 0.35 | 0.268 | 0.36 | | 0.35 | 0.185 | 0.35 | | 0.36 | 0.414 |

Notes: * indicates *p* < 0.05
